# Viral genetic variation accounts for a third of variability in HIV-1 set-point viral load in Europe

**DOI:** 10.1371/journal.pbio.2001855

**Published:** 2017-06-12

**Authors:** François Blanquart, Chris Wymant, Marion Cornelissen, Astrid Gall, Margreet Bakker, Daniela Bezemer, Matthew Hall, Mariska Hillebregt, Swee Hoe Ong, Jan Albert, Norbert Bannert, Jacques Fellay, Katrien Fransen, Annabelle J. Gourlay, M. Kate Grabowski, Barbara Gunsenheimer-Bartmeyer, Huldrych F. Günthard, Pia Kivelä, Roger Kouyos, Oliver Laeyendecker, Kirsi Liitsola, Laurence Meyer, Kholoud Porter, Matti Ristola, Ard van Sighem, Guido Vanham, Ben Berkhout, Paul Kellam, Peter Reiss, Christophe Fraser, BEEHIVE collaboration

**Affiliations:** 1 Department of Infectious Disease Epidemiology, Imperial College London, London, United Kingdom; 2 Big Data Institute, Li Ka Shing Centre for Health Information and Discovery, Nuffield Department of Medicine, University of Oxford, Oxford, United Kingdom; 3 Laboratory of Experimental Virology, Department of Medical Microbiology, Center for Infection and Immunity Amsterdam (CINIMA), Academic Medical Center of the University of Amsterdam, Amsterdam, the Netherlands; 4 Wellcome Trust Sanger Institute, Wellcome Genome Campus, Hinxton, Cambridge, United Kingdom; 5 Stichting HIV Monitoring, Amsterdam, the Netherlands; 6 Department of Microbiology, Tumor and Cell Biology, Karolinska Institutet, Stockholm, Sweden; 7 Department of Clinical Microbiology, Karolinska University Hospital, Stockholm, Sweden; 8 Division for HIV and other Retroviruses, Robert Koch Institute, Berlin, Germany; 9 School of Life Sciences, Ecole Polytechnique Fédérale de Lausanne, Lausanne, Switzerland; 10 Swiss Institute of Bioinformatics, Lausanne, Switzerland; 11 HIV/STI reference laboratory, WHO collaborating centre, Institute of Tropical Medicine, Department of Clinical Science, Antwerpen, Belgium; 12 Department of Infection and Population Health, University College London, London, United Kingdom; 13 Department of Epidemiology, John Hopkins University, Baltimore, Maryland, United States of America; 14 Department of Infectious Disease Epidemiology, Robert Koch-Institute, Berlin, Germany; 15 Division of Infectious Diseases and Hospital Epidemiology, University Hospital Zurich, Zurich, Switzerland; 16 Institute of Medical Virology, University of Zurich, Zurich, Switzerland; 17 Department of Infectious Diseases, Helsinki University Hospital, Helsinki, Finland; 18 Laboratory of Immunoregulation, National Institute of Allergy and Infectious Diseases, National Institutes of Health, Baltimore, Maryland, United States of America; 19 Department of Health Security, National Institute for Health and Welfare, Helsinki, Finland; 20 INSERM CESP U1018, Université Paris Sud, Université Paris Saclay, APHP, Service de Santé Publique, Hôpital de Bicêtre, Le Kremlin-Bicêtre, France; 21 Virology Unit, Immunovirology Research Pole, Biomedical Sciences Department, Institute of Tropical Medicine, Antwerpen, Belgium; 22 Kymab Ltd, Cambridge, United Kingdom; 23 Division of Infectious Diseases, Department of Medicine, Imperial College London, London, United Kingdom; 24 Department of Global Health, Academic Medical Center, Amsterdam, the Netherlands; Universitat de Valencia Institut Cavanilles de Biodiversitat i Biologia Evolutiva, Spain

## Abstract

HIV-1 set-point viral load—the approximately stable value of viraemia in the first years of chronic infection—is a strong predictor of clinical outcome and is highly variable across infected individuals. To better understand HIV-1 pathogenesis and the evolution of the viral population, we must quantify the heritability of set-point viral load, which is the fraction of variation in this phenotype attributable to viral genetic variation. However, current estimates of heritability vary widely, from 6% to 59%. Here we used a dataset of 2,028 seroconverters infected between 1985 and 2013 from 5 European countries (Belgium, Switzerland, France, the Netherlands and the United Kingdom) and estimated the heritability of set-point viral load at 31% (CI 15%–43%). Specifically, heritability was measured using models of character evolution describing how viral load evolves on the phylogeny of whole-genome viral sequences. In contrast to previous studies, (i) we measured viral loads using standardized assays on a sample collected in a strict time window of 6 to 24 months after infection, from which the viral genome was also sequenced; (ii) we compared 2 models of character evolution, the classical “Brownian motion” model and another model (“Ornstein–Uhlenbeck”) that includes stabilising selection on viral load; (iii) we controlled for covariates, including age and sex, which may inflate estimates of heritability; and (iv) we developed a goodness of fit test based on the correlation of viral loads in cherries of the phylogenetic tree, showing that both models of character evolution fit the data well. An overall heritability of 31% (CI 15%–43%) is consistent with other studies based on regression of viral load in donor–recipient pairs. Thus, about a third of variation in HIV-1 virulence is attributable to viral genetic variation.

## Author summary

The severity of the outcome of infection by a pathogen depends on many distinct factors. These include the environment and the genetic sequences of both the host and the pathogen, among others. The fraction of variability in disease outcome explained by pathogen genetic factors is termed “heritability”, because these factors are “inherited” by the new host upon infection. Quantifying heritability is key to understanding the development of the disease and the evolution of the virus. Here, we determined heritability of set-point viral load (SPVL) in HIV-1. SPVL is the stable value of viraemia in asymptomatic infection and it is a strong predictor of disease severity. While heritability of SPVL has been estimated using comparisons of viral genome sequences, this has resulted in widely variable estimates of heritability. Using a large dataset of patients living in Europe, standardised viral loads measures, and new methods, we obtain a more definitive estimate of HIV-1 SPVL heritability in Europe at about 30%. Thus, a significant amount of the variation in disease outcome is explained by the genetics of the virus.

## Introduction

The outcome of infection by the human immunodeficiency virus-1 (HIV-1, henceforth “HIV” for simplicity) is highly variable across individuals, with time to AIDS ranging from 2 years to more than 20 years [1–4]. Quantifying the fraction of this variability explained by genetic variability in the virus is important to our understanding of the mechanisms of pathogenesis and of the evolution of virulence [5].

Most studies have focused on set-point viral load (SPVL), which is a robust predictor of time to AIDS [6]. Following HIV infection, viraemia rapidly increases and reaches a peak before dropping precipitously once HIV-specific cytotoxic T-cells are produced by the immune system [7,8]. After this transient peak has passed, about a month after infection, the subsequent viraemia is much more stable [8] (although it slowly increases over the course of untreated infection [9]). This relatively stable value defines the SPVL.

The extent to which SPVL is determined by the viral genotype—the heritability of SPVL—is defined as the fraction of phenotypic variance in SPVL attributable to variability in viral genotypes [10–12]. The total variance in SPVL in the population may emerge from genetic variation in the host, variation in the immune response, variation because of measurement error, and variation in viral genotypes. Heritability estimates the contribution of the latter. Heritability will determine the statistical power and necessary sample size to find viral molecular determinants of virulence (for example, in genome-wide association studies [13]). Heritability also determines the rate at which viral populations can evolve in response to selective pressures. For example, it has been suggested that an intermediate value of SPVL maximizes viral fitness, because higher SPVL translates into higher transmission rates but shorter disease duration [14]. The rate at which HIV evolves to this optimal value depends on the heritability of SPVL [15,16]. Alternatively, SPVL may change over time not because it is directly under selection but because viral mutations that indirectly affect SPVL are under selection—for example, selection for immune escape or drug resistance. These ideas are not just theoretical possibilities: recent meta-analyses have shown that the population mean SPVL varies over time in many settings [17,18]. For example, in Europe, the mean SPVL of individuals who seroconverted at the beginning of the 1980s was 10,000 copies/mL, whereas it is 30,000 copies/mL for those who seroconverted at the beginning of the 2000s [18]. In contrast, in Botswana, it has been hypothesised that a decline in set-point viral loads was caused by the evolution of costly cytotoxic T lymphocyte (CTL) escape mutations [19]; in Uganda, a decline in set-point viral loads was explained by viral adaptation to a low optimal value under a transmission–virulence trade-off [16]. As well as being a predictor of disease progression, viral load is also a strong predictor of transmission [20]: as a result, spatiotemporal variation in population SPVL will affect epidemic trends. Thus, it is of both biological and public health interest to assess if trends in SPVL can be explained by viral evolution.

Despite many studies into the determinants of virulence, estimates of the fraction of variability in SPVL attributable to genetic variability in HIV vary greatly. Heritability can be estimated in 2 ways. Firstly, as the regression coefficient of recipient SPVL onto donor SPVL in a set of transmission pairs. This is exactly analogous to the classical parent–offspring regression used in quantitative genetics [10,11]. With this method, heritability of SPVL has been estimated to be 33% (95% confidence interval 20% to 46%) in a meta-analysis of studies from sub-Saharan Africa including 433 couples [21–23] (reviewed in [5]). Secondly, heritability can be estimated from SPVL measurements and phylogenetic relationships between viruses (the “phylogenetic mixed model” [24]). This comparative approach is based on the assumption that the covariance between the SPVL of 2 individuals is proportional to their shared ancestry on the viral phylogeny [24,25]. This assumption holds if SPVL evolution can be described by Brownian motion (BM); the phylogenetic mixed model, and methods based on summary statistics such as Pagel’s lambda [26] or Blomberg’s K [27], were all developed under this assumption. Estimation of heritability of SPVL using such comparative methods have yielded more variable estimates of heritability: from 0% to around 60% in Switzerland depending on the subset of the data [12,28]; no significant heritability in Uganda and the Netherlands [28]; and 6% (CI 3%–9%) in the United Kingdom [29]. Thus, no consistent picture has emerged from these various reports.

Inconsistency between estimates may be caused by genuine differences in heritability across populations, limited sample size, uncontrolled variability in SPVL, or limitations in the methods. Heritability may vary across populations and over time because it depends on the genetic variability present in the viral population. For example, one may expect heritability of SPVL to be larger in sub-Saharan Africa, where the viral population is very genetically diverse and multiple subtypes are present [30,31], than in Europe or North America, where the viral population is dominated by subtype B [32]. Moreover, any uncontrolled source of environmental variation—such as variability in the assays used to quantify viraemia, host variability, or intermittent coinfections increasing HIV replication—decreases heritability. This may explain why heritability was highest in the most homogeneous and strictly defined subset of individuals in the Swiss HIV cohort study [12].

The methods used to measure heritability also have several limitations. Methods based on donor–recipient regression do not rely on assuming a specific model of evolution of SPVL, but they can only be applied to datasets that consist of transmission pairs and thus are not suited to estimating heritability for large populations or in a wide range of settings. In addition, genetic confirmation of these transmission pairs is important to avoid downward-biased estimates of heritability [21]. Phylogenetic methods typically include more data and can be applied to more settings, because they use the SPVL measures of a whole cohort (not only transmission pairs), together with the inferred phylogenetic relationships between viral samples [24,25]. This comes at the cost of making specific assumptions on the model of SPVL evolution (for example, the BM model is often assumed [12,29]). The BM model can be interpreted as random unconstrained neutral evolution of virulence, defined as an intrinsic but unknown property of the virus that influences SPVL. In this model, when a patient is infected, the patient’s SPVL is determined both by the virulence of the virus and by other external factors, such as host genotype, dynamics of the host immune response, environmental factors, random effects, etc. The change in virulence over a time step Δt is drawn from a normal distribution with mean 0 and stochastic variance proportional to Δt. Thus, changes in virulence are random and independent from one time step to the next, resulting in no sustained directional trend in virulence and a constant increase in the genetic variance of virulence (and so SPVL) over time. These features may be considered unrealistic, as multiple selective pressures may act upon HIV virulence [14,33], and directional trends in SPVL are observed in some cohorts [17,18]. When virulence evolves under selection, inference of heritability under BM is biased, usually downwards [34,35]. Similarly, phylogenetic methods have little power to detect heritability when the evolution of virulence does not follow a BM model [28].

We hypothesised that inconsistency between estimates in phylogenetic studies of SPVL heritability may have been caused by 5 factors: different viral genetic variance between settings, limited sample size, heterogeneous SPVL measures and definitions, additional noise because of imperfectly estimated phylogenies, and inappropriate use of the Brownian motion model of character evolution. To overcome these limitations and understand what factors caused inconsistencies, we measured heritability of SPVL in a large European cohort collaboration (*N* = 2,028). We obtained blood samples of seroconverters from Belgium, France, the Netherlands, Switzerland, and the UK. Using a cohort of seroconverters allowed us to control for the time since infection, which is associated with viral load because of time trends within patients. We inferred heritability using a classical definition of SPVL, namely the mean (after a log_10_ transformation) of all viral loads measured between 6 and 24 months after infection. Averaging multiple viral load measurements from different time points reduces error variance but may also average out biologically important fluctuations in viral genotype or phenotype within the patient. We also remeasured viraemia using a standardized choice of assay on a single sample taken between 6 and 24 months after infection and before antiretroviral therapy was started (“gold standard viral load”, GSVL), and we reconstructed the viral genome from the same sample. The controlled time window of 6 to 24 months limits variability because of the stage of the infection for both the SPVL and GSVL measures. Additionally, the GSVL measure was defined to limit spurious variation because of variability in assays, to more closely link the viral genotype and phenotype by measuring them from the same sample and to expose variation because of within-host fluctuations in genotype and phenotype that is averaged out in SPVL measures. Henceforth, we use the term ‘viral load’ to mean either SPVL (as classically defined) or GSVL. To improve the resolution of our inferred phylogenies, we generated whole viral genomes rather than genotypes based on the polymerase (*pol*) gene as in previous studies. To avoid the limitations associated with an inappropriate model of character evolution, we compared the classical phylogenetic mixed model based on BM to the Ornstein–Uhlenbeck (OU) model, which includes stabilising selection on viral load. The OU model has been widely used in comparative biology since its introduction in the 1990s [36]. Recently, the OU model was argued to be more appropriate than the BM model to quantify heritability of viral traits [34] and was applied to several datasets ([35] and the present study).

Using a large dataset, carefully measured viral load, whole-genome HIV sequences, and new methods, we showed that the heritability of GSVL is 31% (CI 15%–43%), and the heritability of SPVL is 21% (CI 10%–36%).

## Results

### Viral load is significantly determined by viral genetic factors

**Fig 1:**
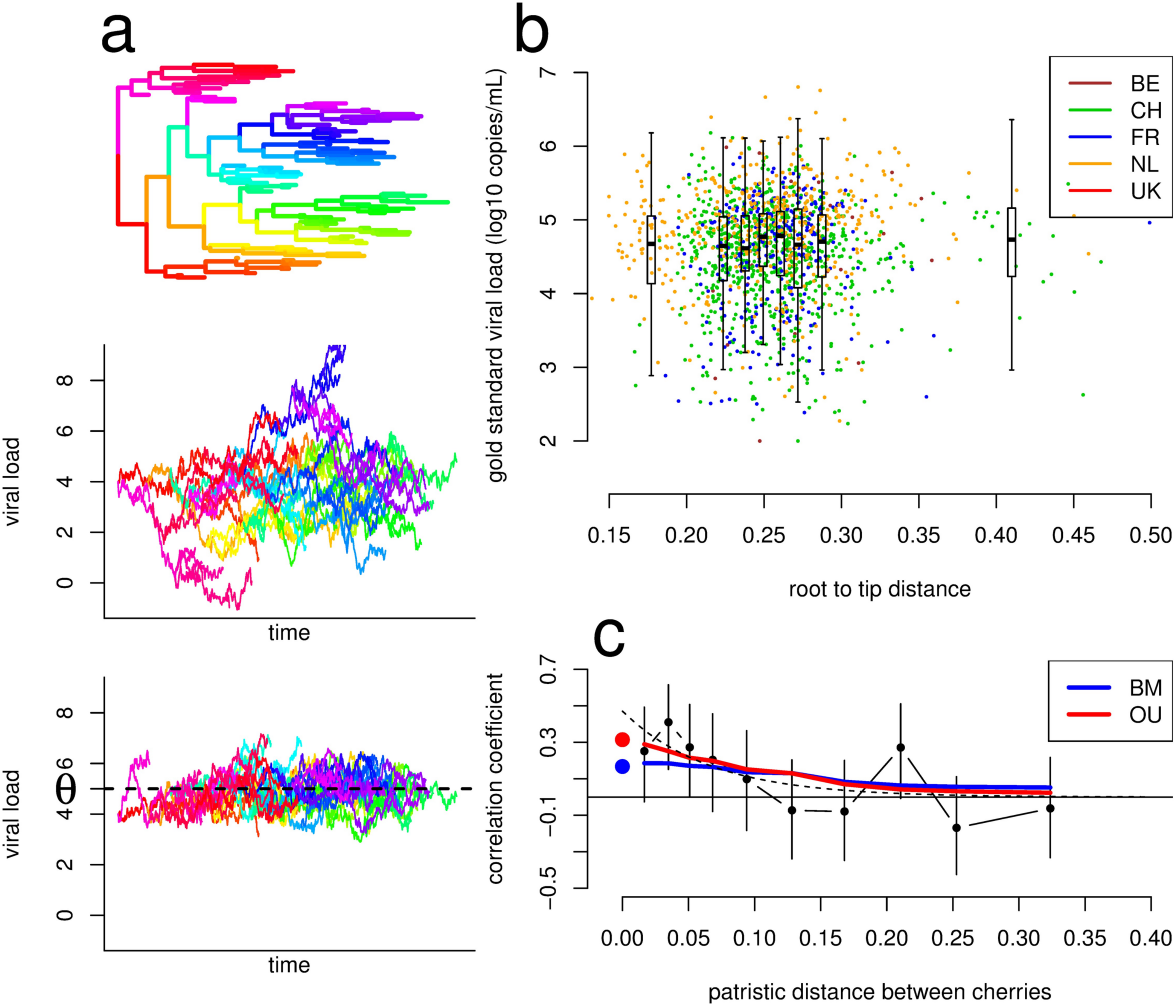
Predictions from the Brownian motion (BM) and Ornstein-Uhlenbeck (OU) models of evolution. **(A)** Illustration of the models of character evolution on a phylogeny (top panel), showing unconstrained neutral evolution leading to increasing genetic variance under the BM model (middle panel) versus stabilizing selection around an optimum θ under the OU model, which results in stable variance over time (bottom panel). Edges of the phylogeny were arbitrarily colored for illustrative purposes. **(B)** The distribution of gold standard viral load (GSVL) over evolutionary time (as quantified by root-to-tip distance [i.e., distance from the common ancestor as assessed by the phylogeny]). Points are the data; boxplots show the median, lower, and upper quartiles, and the whiskers are the lower and upper quartile minus or plus 1.5 times the interquartile range for 8 bins of equal size. **(C)** The correlation coefficient of GSVL across 511 phylogenetic cherries in the subtype B phylogeny as a function of the patristic distance between cherries. Phylogenetic cherries were grouped by patristic distance in 10 bins of equal size. Points are the data, the dashed line is a decreasing exponential fit on the data, and thick lines show predictions from the maximum likelihood (ML) BM and OU models. The large points at patristic distance 0 show the population-level heritability estimated under the BM (blue) and OU (red) model. The data used in the figure are provided as S1 Data.

We fitted the following 3 stochastic models, presented in order of increasing complexity. (i) A null model with only a random component uncorrelated across the tips of the phylogeny, implying zero heritability. (ii) The BM model in which viral load evolves randomly along the branches of the tree, leading to unconstrained increases in genetic variance over time—the model most commonly used in comparative studies [24,25]. (iii) The OU model, which includes a random component similar to BM as well as stabilising selection that brings viral load towards an optimal value and maintains variance at a stochastic equilibrium level [36] (Fig 1A, Materials and methods). Both BM and OU, like all common models of character evolution on phylogenies, assume that evolution occurs continuously on the branches of the phylogenetic tree and make no distinction between within-host and between-host evolution.

The consequences of these models of character evolution on viral load can be described as a linear model with a fixed effect representing the expectation of viral load at the tips and a random effect with a covariance structure depending on the phylogenetic tree and the model of character evolution (Materials and methods). We also adjusted for the confounding covariates sex, transmission mode, age, ethnicity, and viral load assay by including them as additional fixed effects in the linear model. If these factors affected the viral load, they would have contributed to the environmental variance in viral load. Failing to adjust for these covariates would have then inflated estimates of heritability if these factors were clustered in the phylogeny. We did not adjust for the covariates “country” and “viral subtype” in the main analysis as they are correlated with viral genotype.

Viral load was either GSVL, a measure of viraemia remeasured on the same sample as used for viral genetics using a standardized choice of assay (Materials and methods), or the traditional SPVL measure, which is classically defined as the mean of log_10_ viral load for samples from multiple longitudinal samples during a defined period after the first HIV-positive test.

The phylogenetic tree was computed from whole-genome sequences reconstructed from short-read next-generation sequence data [37] and stripped of positions associated with previously identified drug-resistant mutations [38,39] and CTL escape mutations [40] (Materials and methods) (S1 Fig). Mutations at these positions are under strong selection and may independently evolve in different branches of the tree, thus leading to incorrect phylogenetic inference. Results were similar when including these mutations (S3 Table).

Models in which viral load is partly determined by viral genetic factors evolving along the phylogenetic tree had a significantly better fit than the null model, but there was no strong support for the OU over the BM model (Table 1, model comparison based on Akaike Information Criterion (AIC), ΔAIC = –19.6 for BM, ΔAIC = –21.4 for OU compared to the null model). The OU model with stabilising selection was more strongly supported in the set of all subtypes.

**Table 1:** Summary of model fit for the subset of subtype B viruses and for all subtypes.

Subtype	Model	Measure	*N*	AIC	AIC weight	V_E_	σ^2^	α	Optimum θ	h^2^
B	NULL	GSVL	1,581	3,461.7	0	0.52 (0.47–0.55)	-	-	-	0 (0–0)
	BM	GSVL	1,581	3,442.1	0.29	0.43 (0.39–0.47)	0.5 (0.22–0.76)	-	-	0.17 (0.08–0.26)
	OU	GSVL	1,581	3,440.3	0.71	0.35 (0.29–0.43)	3 (0.96–4.8)	8.5 (2.4–10)	4.4 (3.7–5.1)	0.31 (0.15–0.43)
	NULL	SPVL	1,581	3,355.6	0	0.48 (0.44–0.51)	-	-	-	0 (0–0)
	BM	SPVL	1,581	3,343.2	0.66	0.42 (0.37–0.46)	0.35 (0.14–0.54)	-	-	0.13 (0.05–0.2)
	OU	SPVL	1,581	3,344.5	0.34	0.37 (0.29–0.42)	1.7 (0.44–3.5)	7.6 (1.2–10)	4.1 (3.5–4.9)	0.21 (0.1–0.36)
	NULL	CD4 slope	1,170	377	0.02	0.08 (0.067–0.096)	-	-	-	0 (0–0)
	BM	CD4 slope	1,170	370.8	0.55	0.07 (0.063–0.08)	0.048 (1e-06–0.088)	-	-	0.11 (0–0.19)
	OU	CD4 slope	1,170	371.3	0.43	0.071 (0.056–0.079)	0.043 (1e-06–0.37)	0.095 (1.1e-06–10)	–4.9 (–520000 to 0.041)	0.1 (0.01–0.27)
All	NULL	GSVL	2,028	4,474.8	0	0.53 (0.49–0.55)	-	-	-	0 (0–0)
	BM	GSVL	2,028	4,463.3	0	0.45 (0.41–0.49)	0.38 (0.19–0.62)	-	-	0.17 (0.09–0.29)
	OU	GSVL	2,028	4,451.6	1	0.35 (0.29–0.43)	3.6 (1.6–5.1)	10 (6.9–10)	4.2 (3.9–4.6)	0.32 (0.18–0.44)
	NULL	SPVL	2,028	4,395.4	0	0.51 (0.47–0.53)	-	-	-	0 (0–0)
	BM	SPVL	2,028	4,388.4	0.08	0.45 (0.42–0.48)	0.3 (0.11–0.44)	-	-	0.14 (0.05–0.22)
	OU	SPVL	2,028	4,383.5	0.92	0.38 (0.32–0.47)	2.6 (0.32–3.6)	10 (3.5–10)	4.2 (3.5–4.7)	0.24 (0.06–0.34)
	NULL	CD4 slope	1,476	471.7	0.01	0.08 (0.069–0.091)	-	-	-	0 (0–0)
	BM	CD4 slope	1,476	464.3	0.45	0.072 (0.065–0.08)	0.033 (0.0016–0.058)	-	-	0.1 (0.01–0.18)
	OU	CD4 slope	1,476	463.9	0.54	0.069 (0.058–0.077)	0.081 (1e-06–0.38)	2.9 (1.6e-06–10)	–0.3 (–170000 to 0.051)	0.13 (0.01–0.25)

Maximum likelihood estimates are given with 95% parametric bootstrap confidence intervals, accounting for uncertainty because of finite sample size and uncertainty in phylogenetic reconstruction (*N* = 100 bootstraps, see text for details). Three phylogenetic models (NULL, BM, and OU) are presented for 3 viral phenotypes: GSVL and SPVL are in units of log_10_ viral copies/mL of blood and CD4 slope is in units of cells/mm^3^/day. *N* is the sample size. AIC is the Akaike Information Criterion; AIC weight for model *i* (NULL, BM, or OU) is defined as $w_{i}=\frac{\text{exp}\left[\left(aic_{i}-\text{min}\left(aic\right)\right)/2\right]}{\sum_{i=1}^{3}\text{exp}\left[\left(aic_{i}-\text{min}\left(aic\right)\right)/2\right]}$. V_E_ is the environmental variance, including host factors and error variance (but not variability in assay, as the linear regression adjusts for assay). σ^2^ is the stochastic variance governing evolution of the viral genetic factors along the phylogeny. α is the strength of stabilising selection in the OU model. The optimum θ is the optimal trait in the OU model. h^2^ is the heritability. BM, Brownian motion; GSVL, gold standard viral load; OU, Ornstein–Uhlenbeck; SPVL, set-point viral load.

### Heritability of GSVL among subtype B samples is 31%

**Fig 2:**
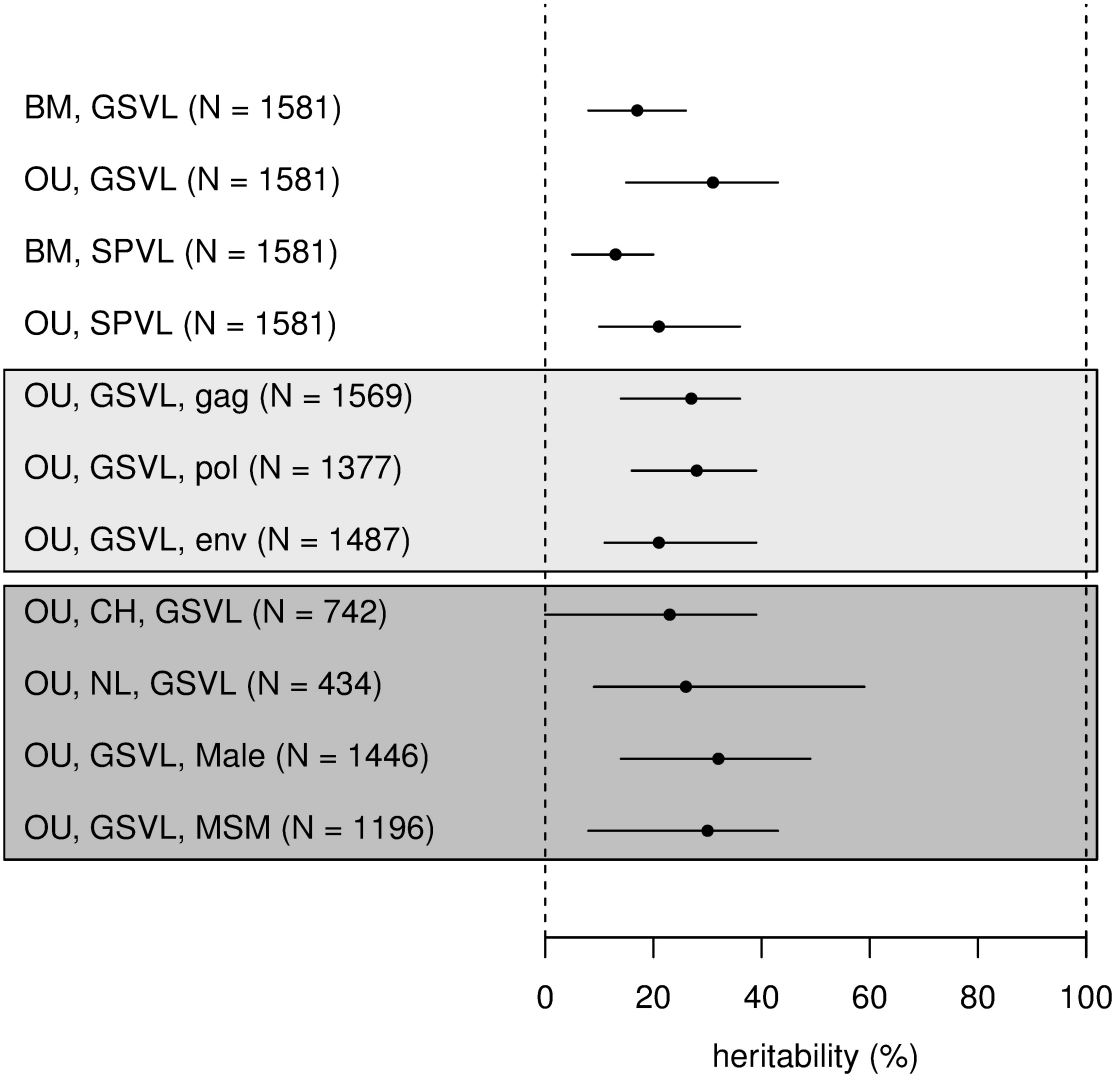
Maximum likelihood estimates of heritability (points) and bootstrap confidence intervals (segments) for subtype B sequence. This is shown for the BM and OU model and the GSVL and SPVL measures of viral load for the whole-genome phylogeny; for the OU model and GSVL on the phylogeny inferred only from *gag*, *pol*, and *env* genes (light grey box); and for several stratifications of the data, only when the size of the subset was greater than 400 (dark grey box). CH: Switzerland, NL: the Netherlands; male sex; MSM. The data used in the figure are provided as S1 Data.

The maximum likelihood (ML) models of character evolution implied heritability for GSVL of 17% [8%–26%] under the BM model and 31% [15%–43%] under the OU model (Table 1, Fig 2). Heritability is defined as the fraction of phenotypic variance explained by genetic variance in a given population. More precisely, we estimated broad-sense heritability: the contribution to phenotypic variance of all genetic variation (including variation generated by epistatic effects between loci). This contrasts with narrow-sense heritability, which quantifies only the contribution of additive effects of individual genetic variants [35]. We estimated heritability by resimulating the ML model of viral load evolution on the tree. A simulation attributed a value of viral load to each tip of the tree as the sum of a viral genetic component and an environmental component (the latter including epidemiological covariates). Heritability was the variance of the genetic component (across tips) divided by the total variance in the simulations. Because the models of evolution are stochastic, each run gave a different value of heritability; therefore, we reported the mean heritability across 1,000 stochastic simulations. We also developed an analytical expression for the expectation of heritability as a function of model parameters that allows faster calculations and proved very accurate (Materials and methods). In accordance with theoretical expectations [35], assuming a BM model of character evolution led to lower heritability than assuming an OU model. The BM model theoretically results in steady expansion of the genetic variance over phylogenetic time. A SPVL variance similar at tips closer to the root and at tips further away from the root goes against this prediction and leads to a downward bias in the estimated genetic variance and heritability. In contrast, the OU model allows high heritability even with a stable variance (Fig 1A). In our data, epidemiological covariates (sex, transmission mode, age, ethnicity, assay) would have inflated heritability by 4% were they not accounted for (heritability of GSVL without adjustment for these covariates was at 21% and 35% under BM and OU), with an effect of sex in particular. This means some of these covariates affected viral load and were clustered in the phylogenetic tree. Specifically, in the subset of subtype B, for GSVL, under the null model, males had +0.3 log_10_ copies/mL higher GSVL than females (CI 0.15–0.45) (type II analysis of variance, *p* = 0.0002). Mode of transmission, age, and ethnicity did not have a significant effect in the subset of subtype B (S4 Table), although mode of transmission had a significant effect when considering all participants (S5 Table), with men having sex with men (MSM) transmission associated with +0.14 [CI 0.046–0.24] log_10_ copies/mL higher GSVL compared to heterosexual transmission. The type of assay had an effect on SPVL (type II analysis of variance, *p* = 0.0019) but no effect on GSVL (*p* = 0.24). This confirms the better standardisation of the GSVL measure. Effects were similar under the BM and OU model. Lastly, including “country” as a covariate in the phylogenetic regression lowered heritability by 5% to 6% (S3 Table). This difference partly represents genuine heritability because the viral genotype is expected to differ by country.

We quantified uncertainty on the parameters and on heritability using parametric bootstrapping on the bootstrapped trees. This method combines uncertainty because of finite sample size and uncertainty in the phylogenetic tree inference. For each of the 100 bootstrap trees, we simulated a stochastic outcome of the ML model, reinferred ML parameters from these simulations, and calculated the confidence intervals on these reinferred parameters. Uncertainty on heritability was large for the OU model, ranging from 15% to 43%, in spite of the large dataset (*N* = 1,581).

Heritability of GSVL was higher than heritability of SPVL. This difference, however, was not due to higher environmental variance for SPVL but rather was due to higher genetic variance for GSVL (environmental variance V_E_ was similar for the 2 measures, but the stochastic variance σ^2^ describing evolution of the genetic component is higher for GSVL, Table 1).

The structuring of the viral population into several subtypes did not contribute much to heritability, as heritability was only slightly higher across all subtypes (Table 1), and we did not detect any effect of subtype on viral load (type II analysis of variance, *p* = 0.65, S5 Table). The interpretation of heritability across all subtypes is difficult, as many of the nonsubtype B sequences were recombinants. This means that in the phylogenetic tree of all subtypes, the topology, and branch lengths between subtypes cannot be interpreted in terms of the amount of evolution on a line of vertical transmission, hindering phylogenetic interpretation. Because of the diversity of non-B viruses in our sample, we did not have sufficient data to individually estimate heritability for specific non-B subtypes.

We next stratified the analysis by country, sex, and mode of transmission. To first estimate the power to detect heritability in smaller subsets of data, we systematically subsampled the main dataset at random and measured maximum likelihood heritability and confidence intervals as a function of sample size (S2 Fig). We found that accurate and precise estimation of heritability required samples of at least 500 individuals, especially when using the OU model of character evolution. Accordingly, we found no significant heritability in most stratifications of the data. Heritability measured separately within each country was significant only in the Netherlands, at 26% (12%–60%) (S2 Table, *N* = 434, ΔAIC = –12.2 for OU compared to the null model). In Switzerland, the largest cohort in this study, GSVL was not significantly heritable (S2 Table, *N* = 742, ΔAIC = +2.1 for OU compared to the null model). This could reflect the limited genetic diversity of our Swiss samples; in other countries, the lack of detected heritability is most likely due to limited power to detect a phylogenetic signal. In males infected by subtype B viruses (*N* = 1,446), GSVL heritability was 16% under BM and 32% under OU, but heritability was not significant in females (*N* = 135). In MSM infected by subtype B viruses (*N* = 1,196), GSVL heritability was 17% under BM and 30% under OU, but heritability was not significant in injecting drug users (IDUs) (*N* = 110) and heterosexuals (*N* = 211).

### Goodness of fit tests

Both BM and OU models fitted the data well. One major difference in the prediction of the 2 models is that in BM, the genetic variance keeps increasing with genetic distance as neutral genetic variation accumulates, whereas in OU genetic variance initially increases and then eventually reaches equilibrium between generation of variation and stabilising selection (Fig 1A). The maximum likelihood BM and OU models both predicted an increasing genetic variance at a rate of +0.0024 log_10_ copies^2^/mL^2^/year for BM and +0.0011 log_10_ copies^2^/mL^2^/year for OU (S3 Fig). The intermediate increase in variance predicted by the OU model means that the distribution of viral loads has not yet converged to its steady state. In our dataset, the average viral load was constant over time, and phenotypic variance increased over time in GSVL at +0.01 log_10_ copies^2^/mL^2^/year but did not significantly increase in SPVL (S1 Table).

As well as predicting the variance, the models predict the covariance structure of the viral loads at the tips of the phylogeny. We assessed goodness of fit on the subset of 511 phylogenetic cherries (pairs of adjacent tips that are each mutually closest to each other) on the tree of subtype B viruses. In addition to testing goodness of fit, focusing on cherries allows phylogenetic approaches to be compared to the donor–recipient regression that are based on classical quantitative genetics [28,35]. Indeed, phylogenetic cherries that are genetically very similar (separated by a small patristic distance) on a phylogenetic tree are more likely to be donor–recipient pairs than genetically distant pairs [41]. We computed the Pearson correlation coefficient between viral load values across cherries, stratified by the patristic distance (the distance between the 2 tips) (Fig 1C). In the limit in which patristic distance is 0, the expected value of both correlation coefficients is equal to heritability (S2 Text, see also [35]). For OU, the correlation decreases with patristic distance. For BM, all else being equal, the correlation should in theory be independent of patristic distance (Materials and methods). However, here we see that for the BM model the correlation decreased with patristic distance, because those pairs of tips separated by a large patristic distance tend to also have less shared ancestry. In accordance with the predictions of both models, the correlation coefficient for GSVL of cherries separated by a small patristic distance was around 30%, not far from the predicted heritability of 17% (under BM) and 31% (under OU) (Fig 1C). The observed negative relationship between the correlation coefficient and the patristic distance resembled closely the prediction from both models (Fig 1C).

### Variation in heritability across the genome

**Fig 3:**
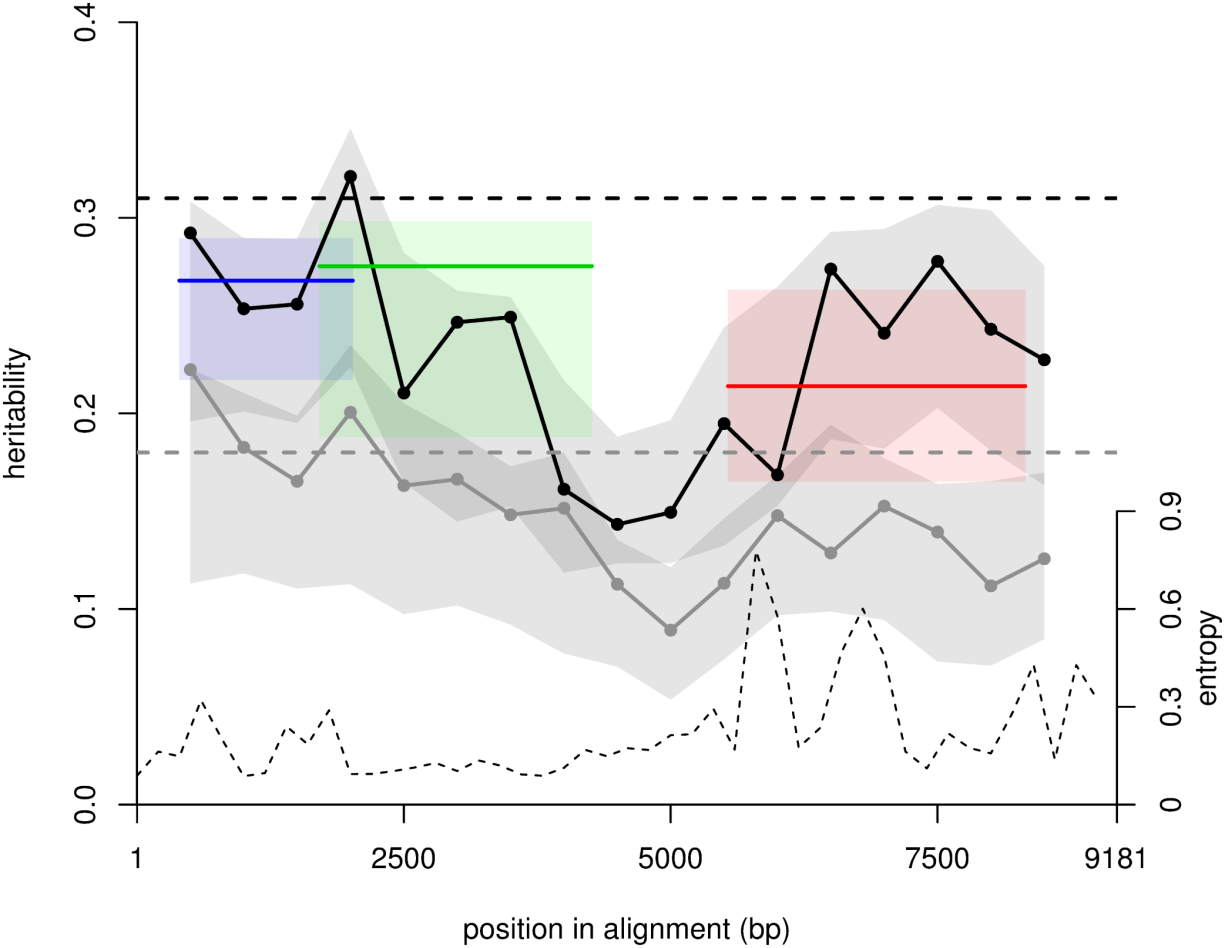
Maximum likelihood estimates of heritability across the genome. Heritability was inferred for overlapping windows of 1,000 bp separated by 500 bp for the Ornstein–Uhlenbeck (OU) model (black bullets) and the Brownian motion (BM) model (grey bullets). The horizontal dashed lines are the whole-genome heritability estimates. The 3 colored segments show heritability for *gag*, *pol*, and *env* genes in blue, green, red (for OU only). Confidence intervals (grey and colored regions) reflect phylogenetic uncertainty. The largest heritability is in the region where *gag* and *pol* overlap. We also show entropy—a measure of genetic diversity—along the genome (dashed curve and right axis). Entropy at a position was calculated as −Σ_*i* ∈ {*A*,*C*,*G*,*T*}_*p*_*i*_ log(*p*_*i*_), and we show the average entropy over 200-bp windows. The data used in the figure are provided as S1 Data.

When using only the phylogenetic information contained in a single gene instead of the whole genome, we found heritability for the OU model was 27% (14%–36%) in the *gag* gene, 28% (16%–39%) in *pol*, and 21% (11%–39%) in *env* (Table 2, Fig 3). The lower heritability for each gene independently compared with the whole genome (h^2^ = 31%) was expected. In the limit of no recombination, the phylogenetic history of all genes is the same: thus, with perfect phylogenetic resolution, heritability is the same across genes and equal to total heritability for the whole genome. At the other limit where the 3 genes evolve independently (linkage disequilibrium between them is 0), the heritability for each gene reflects the contribution of molecular variation at that gene on total variation, and whole-genome heritability is the sum of heritability across genes. Indeed, if the viral load can be written *v* = *g*_*gag*_ + *g*_*pol*_ + *g*_*env*_ + *e* where the terms in *g* denotes the additive genetic contributions of each gene and *e* denotes the effect of the environment, then fitting a model of character evolution on the *gag* tree estimates *g*_*gag*_, while *g*_*pol*_ + *g*_*env*_ is subsumed in *e* because this quantity is randomly distributed on the *gag* tree by the assumption of linkage equilibrium.

**Table 2:** Summary of model fit for GSVL, for subtype B, for each gene. Parameters as in Table 1.

Model	Gene	*N*	AIC	AIC weight	V_E_	σ^2^	α	Optimum θ	h^2^
NULL	*gag*	1,569	3,378.7	0	0.5 (0.46–0.53)	-	-	-	0 (0–0)
BM		1,569	3,351.5	0.14	0.41 (0.37–0.44)	0.9 (0.48–1.3)	-	-	0.17 (0.09–0.26)
OU		1,569	3,347.8	0.86	0.36 (0.31–0.42)	3.2 (1.4–4.5)	10 (3.9–10)	4.3 (3.7–5)	0.27 (0.14–0.36)
NULL	*pol*	1,377	2,814.2	0	0.45 (0.41–0.48)	-	-	-	0 (0–0)
BM		1,377	2,790.5	0.34	0.36 (0.32–0.42)	0.72 (0.29–1.1)	-	-	0.19 (0.08–0.28)
OU		1,377	2,789.2	0.66	0.32 (0.26–0.36)	2.2 (0.83–3.8)	7.2 (1.4–10)	4.3 (3.6–5.1)	0.28 (0.16–0.39)
NULL	*env*	1,487	3,016.3	0	0.44 (0.39–0.47)	-	-	-	0 (0–0)
BM		1,487	2,996	0.56	0.38 (0.32–0.42)	0.24 (0.085–0.42)	-	-	0.13 (0.04–0.23)
OU		1,487	2,996.5	0.44	0.34 (0.25–0.39)	0.87 (0.3–3.4)	3.8 (0.87–10)	4.3 (3.5–5)	0.21 (0.11–0.39)

AIC, Akaike Information Criterion; BM, Brownian motion; OU, Ornstein–Uhlenbeck.

At a finer scale, when inferring the heritability across the genome in 17 overlapping windows that are 1,000 base pairs in length separated by 500 base pairs, we found accordingly that heritability was almost always lower than for the whole-genome inference. Heritability was highest around the region where the *gag* and *pol* genes overlap and lowest in the region between the *pol* and *env* genes, including the *vif*, *vpr*, and *vpu* genes (Fig 3). Linkage disequilibrium dropped rapidly with genetic distance and was small when the distance was greater than 100 bp (S4 Fig). Confidence intervals reflecting phylogenetic uncertainty (Materials and methods) do not overlap between the low- and high-heritability regions. These observations suggest 2 distinct island contributions to heritability, 1 from the low-diversity region coding for the replication machinery (*gag*–*pol*) and the second from the high-diversity region coding for the envelope gene.

### Heritability of the CD4 cell count slope is 11%

Lastly, we investigated the heritability of another viral phenotype, the CD4 cell count slope (the rate at which the patient’s CD4 cell count declines). CD4 slope was not found to be heritable in a previous analysis [12]. We computed the CD4 slope for *N* = 1,476 patients with at least 5 CD4 measures after the date of the first positive HIV test and before antiretroviral therapy. Models in which CD4 slope was partly determined by viral genotype evolving along the phylogeny were favoured over the null model (Table 1, ΔAIC = –6.2 for BM, ΔAIC = –5.7 for OU compared to the null model). Heritability was weak, at 11% [0%–19%] in the favoured BM model (the ML OU model has weak stabilising force, and the inferred heritability was similar to that of the BM model). The coefficient of determination of the relationship between CD4 slope and viral load was R^2^ = 5.2% for GSVL, R^2^ = 7.4% for SPVL. This weak relationship is similar to that found previously [42,43] and may partly be explained by the noisiness of the CD4 slope measure and the difference between viral load and CD4 count in the blood versus in the whole body.

## Discussion

Using a large dataset of whole-genome HIV sequences (*N* = 2,028) from patients with a known seroconversion date with carefully measured viral load, we established that viral genetic factors account for 20% to 30% of variation in viral load in Europe. This estimate of heritability is consistent with those obtained with donor–recipient regression (around 30% [5]), unlike results of previous phylogenetic studies [12,29]. It agrees with a recent analysis performed on 8,483 patients with *pol* sequences from the UK, reporting a lower bound for heritability of SPVL at 25% [35]. We hypothesized that the large variation across previously published phylogenetic estimates could be due to genuine biological differences across cohorts, limited sample sizes, less rigorous selection criteria for patient inclusion leading to heterogeneous viral load measures, less well resolved phylogenies, and/or use of methods based on inappropriate models of character evolution. We found an effect of limited sample sizes and the model of character evolution. However, there was little evidence for genuine biological differences across cohorts included in this data.

This study provides several methodological recommendations for the estimation of heritability. Firstly, precise and accurate estimation of heritability required a sample of at least 500 individuals in our cohort (S2 Fig). Limited sample sizes can generate substantial heterogeneities across estimates, in accordance with previous results showing limited power to detect heritability in existing datasets, and large confidence intervals [28]. Secondly, we compared 2 models of character evolution: BM and OU (the latter including stabilising selection). The BM model of evolution was used in most previous studies of HIV viral load heritability, while the OU model was only recently applied to HIV data ([35] and the present study). In this dataset, we found the 2 models were almost equally supported for subtype B, and the OU model was more supported for all subtypes combined. OU implied higher heritability than BM. Support for the OU model was stronger in the set of all subtypes (Table 1). Because the tree of all subtypes spans a larger phylogenetic distance than the tree of subtype B (the root-to-tip distance is almost twice as large), the BM model fits less well the covariation of viral load between tips and the observed phenotypic variance. In general, we suggest the OU model may better describe viral load evolution for 3 reasons. (i) The OU model, with stabilising selection, is a better fit on previously analysed data from the UK [29] and Switzerland [12], leading to consistent estimates of heritability at about 30% in both cohorts [35]. (ii) Data on 56 donor–recipient pairs show that the correlation coefficient between viral loads of the donor and that of the recipient decreases as the recipient viral load is measured later in infection [44]. This is in accordance with the OU model, in which stabilising selection progressively erases the correlation between donor and recipient viral loads. But it is not in accordance with the BM model, in which the correlation coefficient does not depend on the time elapsed between donor and recipient but only on the amount of evolutionary ancestry that they share. (iii) The OU model, unlike the BM model, is compatible with the hypothesis that viral load is under a transmission–virulence trade-off favouring those viruses giving intermediate viral loads [14]. Under this trade-off, viral load is under stabilising selection as in the OU model, because very low viral loads (resulting in very low transmission rates) and very high viral loads (resulting in very short duration of infection) do not allow much onward transmission of the virus, such that viral load cannot drift unconstrained to very low or very high values. Consistent with this hypothesis, we found that the optimal GSVL was 4.4 log_10_ copies/mL in subtype B viruses (CI [3.5–5.2]), an estimate close to the optimum inferred using epidemiological data [14,16]. Unfortunately, although the OU model is probably more biologically realistic, one must keep in mind that with this model, any temporal trend in viral load will be interpreted as caused by evolution of viral genetic factors. For example, in our dataset (in which SPVL did not significantly change over time), adding a temporal trend of +0.02 log_10_ copies/mL/year (comparable to what has been observed in various European and North American cohorts [17,18]) resulted in the OU model being much more favoured (ΔAIC = 12 compared to the BM model). The BM model is unlikely to result in a sustained temporal trend, especially when the sample size is large. Yet, a temporal trend in viral load may be due to uncontrolled environmental factors—for example, a changing prevalence of coinfections [45] or variability in the viraemia assays—and not necessarily to viral evolution. In a dataset presenting a significant temporal trend in which OU is the best model, the dataset may be analysed with the temporal trend removed to check that the correlation structure of viral load also corresponds best to the OU model. Thirdly, our results reconcile estimation of heritability based on donor–recipient pairs (which consistently estimate heritability at around 30%) and those based on phylogenetic analysis. If we assume the phylogenetic cherries separated by a small patristic distance are indeed sampled from donor–recipient pairs, the heritability estimated here by donor–recipient regression was similar to the prediction based on the phylogenetic analysis (Fig 1C). We also derive formal links between donor–recipient regressions and phylogenetic models (S2 Text).

In this large European cohort, there was no evidence that heritability differed between countries. Given the small sample sizes for each country, we had limited power to detect such differences. However, the similar phenotypic variance across countries and the fact that sequences from different countries are interspersed in the tree also suggested genetic variance in viral load was similar across countries. We found significant heritability only in the Netherlands—26% (*N* = 434, S2 Table). We found no significant heritability in the 742 subtype B sequences from Switzerland (S2 Table). Previous studies have estimated heritability of SPVL in Switzerland at around 60% but only when focusing on the subset of MSM patients with at least 3 viral load measures with little fluctuations across measures [12,28], a subset accounting for 20% of all the data; no significant heritability was found when using all of the data. The limited number of samples from the UK (*N* = 87, S2 Table) made it difficult to compare our results with a previous study finding a heritability of 6% in the UK [29].

We estimated the heritability of 3 viral phenotypes: the single viral load GSVL measured in a standardised way, SPVL, and the rate of CD4 decline. GSVL had higher genetic variance than SPVL, resulting in heritability of 31% for GSVL compared to 21% for SPVL, and we suggest this is due to the closer link between the viral sequence and the phenotype, both obtained from the same sample (S1 Text). However, SPVL was a better predictor of CD4 decline, as it explained R^2^ = 7.4% of the variance in CD4 decline, while GSVL explained R^2^ = 5.2%. This is perhaps not surprising: CD4 decline and SPVL are both temporal averages summarising virulence over a period of time, whereas GSVL is a single measurement typically taken at the beginning of infection (median time between date first positive and sample = 266 days). Note though that assay variability significantly contributed to variation in SPVL (S4 Table), showing the importance of adjusting for assay type when estimating heritability of SPVL. In our definition of heritability, assay error and variability in assays contributed to the denominator (total phenotypic variance). Yet, this variation does not correspond to biological variation in the true trait but to variation caused by imperfect measurement. In principle, we should remove this source of variance from the denominator to obtain the phenotypic variance in the true trait, and this will result in higher heritability. This effect would be negligible for assay variability, which represents only a small fraction of the total variance (S4 Table). Similarly, a plausible value for assay error at 0.04 log_10_ copies^2^/mL^2^ (Materials and methods) would result (with a phenotypic variance of 0.54 log_10_ copies^2^/mL^2^ and heritability at 31%) in a slightly higher heritability of 0.31 x 0.54 / (0.54–0.04) = 33%. Lastly, 11% of variation in the rate of CD4 cell count decline was explained by viral genetic variation. The small correlation between CD4 decline and viral load (around 5%), the CD4 decline heritability of 11%, and the viral load heritability of 31% can be explained by the existence of several classes of viral variants: (i) variants causing more intense exploitation of CD4 cells resulting in faster CD4 depletion and greater viral load, contributing to the heritability of CD4 decline and of viral load; (ii) variants increasing viral load without intensifying the exploitation of CD4 cells, contributing to the heritability of viral load only; and (iii) variants intensifying the exploitation of CD4 cells without increasing viral load, contributing to the heritability of CD4 decline only.

Both models of character evolution assume an additive effect of the viral genotype and an “environment” effect (including host factors). A limitation of this study is that host genotype data was not available, in particular for the host class I HLA alleles, and therefore we could not control for this important factor [46] in the regression. Moreover, variability in viral load may depend on interactions between viral and host genetic factors [13,47], in particular between host class I HLA alleles and viral CTL epitopes. Our model assumes additive contributions of the host and the virus and would force the (host genotype) x (viral genotype) interaction variance into the 2 additive components. This calls for the development of new methods to measure the fraction of variance determined by virus–host interactions [47].

The confirmation that a significant fraction of variability in viral load is determined by viral genetic factors motivates searching for individual viral genetic variants responsible for variation in viral load. The finding of 2 distinct islands’ contributions to heritability, 1 from the replication machinery (*gag* and *pol* genes) and the second from the envelope gene, will guide this search. A previous viral genome-wide association study did not find any significant viral genetic variation associated with viral load [13] but was only powered to detect individual effects accounting for 4% or more heritability. This suggests that the effects of individual variants are small, requiring large sample sizes for their discovery. Viral load may also be determined by epistatic effects between mutations, the detection of which would require even larger sample sizes. Knowledge of these molecular variants would allow us to relate changes in frequency of these variants to the observed temporal trends in viral load [17–19], and most importantly, would provide new insights into HIV pathogenesis, a fascinating but challenging task [5].

In conclusion, around 30% of variation in viral load was explained by viral genetic variation in these European cohorts. This study highlights the need for large datasets, comparison between different models of character evolution, and goodness of fit tests to consistently estimate heritability of viral load in HIV-1 infection and so explains and resolves the inconsistency in previously published estimates.

## Materials and methods

### Patient population

We selected HIV-positive seroconverters from the Antwerp cohort in Belgium (BE), the Swiss HIV Cohort Study in Switzerland (CH), the ANRS PRIMO Cohort in France (FR), the ATHENA cohort in the Netherlands (NL), and the UK register of seroconverters in the UK—all part of the BEEHIVE (“Bridging the Evolution and Epidemiology of HIV in Europe”) collaboration. These were selected as the set of all patients meeting the study’s inclusion criteria, for which we had complete clinical and virus genetic data at the time of analysis. The inclusion criteria were the following. (i) Participants were seroconverters (i.e., the first positive test was less than 1 year after the last negative test), or the participant presented with evidence of recent infection (laboratory evidence or seroconversion illness), ensuring the date of infection was known precisely. (ii) No antiretroviral therapy was taken in the first 6 months following the first positive test. (iii) At least 1 viral load or 1 sample from which viral load can be determined was taken between 6 and 24 months following the first positive test. (iv) At least 1 sample of at least 500 μL of frozen EDTA plasma or serum was taken between 0 and 24 months following the first positive test while antiretroviral therapy (ART)-naive. We also collected information on age, sex, and mode of transmission. All patients consented to this study. All studies within the cohorts were approved by in-country institutional review boards, and the overall BEEHIVE study, which only accessed anonymised data, was approved by the ethics panel of the European Research Council.

### Viral load measures

The stable value of viraemia after acute infection and before the onset of AIDS (our phenotype of interest) was calculated in 2 ways. First, the viral load was remeasured in a standardized way, on a single blood sample taken more than 6 months and less than 24 months after the first positive HIV test and before the start of ART. If viral load had been previously measured with 1 of 3 assays (COBAS AmpliPrep/COBAS TaqMan HIV-1 Test, v2.0 from Roche; Abbott RealTi*M*e HIV-1 Assay from Abbott; Quantiplex HIV-1 RNA Assay, version 3.0 from Chiron Diagnostics, Emeryville, CA), on the same visit when the sample used to determine the viral sequence was taken, we did not repeat the assay. Otherwise, viral loads were repeated with COBAS AmpliPrep/COBAS TaqMan HIV-1 Test, v2.0 on the same sample used to determine the viral sequence. We defined the GSVL as log_10_ of the single viral load (in copies per mL) measured in this way. In several cases, the GSVL was below detection limit, which may be due to genuinely low viraemia or assay failure, but the assay could not be repeated because of material shortage. We eliminated from the analysis the undetectable GSVL values with SPVL greater than 3 log_10_ copies/mL (*N* = 1 value eliminated). The error variance of the assay used for GSVL is between 0.01 log_10_ copies^2^/mL^2^ (standard deviation of 0.1 copies/mL estimated in a standardised way using aliquots of the same sample [48]) and 0.25 log_10_ copies^2^/mL^2^ (standard deviation of <0.5 log_10_ copies/mL estimated from the replicated viral load measures for problematic samples in this study). An intermediate value of 0.04 log_10_ copies^2^/mL^2^ is plausible.

Second, we used the series of viral loads previously measured on the same patients to define the set-point viral load (SPVL), calculated as the average log_10_ (viral load, in copies per mL) for all viral load measurements available between 6 and 24 months after the first positive HIV test (using different assays).

For both the GSVL and the SPVL measures, we adjusted for the potential impact of assay type on viral load within the phylogenetic regression.

### CD4 count measures

Additionally, we quantified virulence of the virus using the rate of CD4 decline in patients. We selected patients who had at least 5 CD4 count measures between the date of the first positive HIV test and the date that ART was first prescribed. We fitted a linear model describing the decline in CD4 count over time within this patient. We recorded the slope of this relationship.

The characteristics of the cohort are summarized in Table 3.

**Table 3:** Summary of the main characteristics of the cohort.

GSVL	Year sampled	Country sampled	Transmission mode	Sex	Age at infection	Ethnicity	Subtype	Sequence length (bp)
Min.: 2.000	Min.: 1985	BE: 52	HEAM/TRANSF: 3	Female: 295	Min.: 17	Black: 36	B: 1,581	Min.: 601
1st Qu.: 4.167	1st Qu.: 2004	CH: 1,005	HET: 475	Male: 1,733	1st Qu.: 29	White: 421	A1: 113	1st Qu.: 7,669
Median: 4.675	Median: 2007	FR: 378	HET/IDU: 44		Median: 35	Other/Unknown: 1,571	02_AG: 107	Median: 9,018
Mean: 4.573	Mean: 2006	NL: 480	IDU: 90		Mean: 36		01_AE: 68	Mean: 8,168
3rd Qu.: 5.066	3rd Qu.: 2009	UK: 113	MSM: 1343		3rd Qu.: 42		C: 41	3rd Qu.: 9,068
Max.: 7.002	Max.: 2015		MSM/IDU: 1		Max.: 78		D: 25	Max.: 9,639
			Other/Unknown: 72		Unknown: 445		(Other): 93	

BE, Belgium; CH, Switzerland; FR, France; GSVL, gold standard viral load; HEAM/TRANSF, haemophiliac or blood transfusion; HET, heterosexual; IDU, injecting drug user; MSM, men having sex with men; NL, the Netherlands; UK, United Kingdom.

### Full genome HIV sequences and phylogeny

Full genome HIV sequences were obtained from blood samples of seroconverters taken between 6 and 24 months after seroconversion and before ART was initiated.

#### Sequencing and assembly

HIV genomes were amplified using a set of universal primers [49], and sequencing was performed using Illumina MiSeq or HiSeq 2500 technology. This generated paired-end reads, which varied in length between 100 and 300 base pairs. HIV genome sequences were assembled for each sample using the custom pipeline ‘shiver’ described in detail elsewhere [37]. In summary, contigs were reconstructed for each sample using the de novo assembler IVA [50]. Non-HIV contigs were removed; remaining contigs were corrected and aligned to standard references. A new reference for mapping was then built for each sample using the contigs, filling gaps with a set of standard whole-genome HIV sequences [51] for positions not covered by the contigs. Reads were mapped to these custom references using SMALT [52]. The consensus base at each position was called, resulting in a single consensus HIV sequence for each sample. A consensus base was called at each position where coverage exceeded 30 reads (for MiSeq technology) or 300 reads (for HiSeq technology). An unambiguous consensus base was called when more than 60% of reads had this base. An ambiguous base as defined by the International Union of Pure and Applied Chemistry (IUPAC) notation was called when the most frequent base was in less than 60% of reads. The programs Trimmomatic [53] and Fastaq [54] were used to trim adapters, PCR primers, and low-quality bases from the reads; BLASTN [55] was used to identify contigs and reads suspected of being contaminants; MAFFT [56] was used to align the contigs to references; and SAMTOOLS [57] was used to process the mapped reads.

We processed the sequences for phylogenetic analysis as follows: we removed positions associated with previously identified drug-resistant mutations [38,39] and CTL escape mutations [40] as well as long terminal repeats. We removed positions where more than 90% of sequences had a gap and removed sequences smaller than 500 base pairs. We inspected the resulting alignment and removed small misaligned sequence fragments. This resulted in an alignment of 1,373 sequences, 75% of which were more than 8,359 base pairs long (Table 3).

#### Subtyping

We subtyped each sequence using the COMET software [58], in particular to identify the subset of subtype B sequences (the dominant subtype in the European HIV epidemic; Table 3). We verified the performance of COMET using the 199 reference sequences of known subtype used in our alignment. COMET correctly attributed the subtype for 177 out of the 199. Almost all subtyping mistakes consisted in imputation of the wrong circulating recombinant form (CRFs). Most relevant to our analysis, which requires classification in B versus non-B subtypes, all 32 subtype B references were correctly classified as subtype B, except 1 that was classified as CRF 12_BF. Conversely, among all non-B references, only 2 were classified as subtype B: CRFs 48_01B and 51_01B (both of which contain subtype B sequences).

In our dataset, a number of sequences were attributed to CRFs 12_BF, 42_BF, or 17_BF. These are most likely mistakes, as these subtypes are not commonly found in Europe. To verify this, we additionally determined subtype by placing these sequences in a phylogenetic tree of the references, using the software RAxML. Subtype was defined as the subtype of the reference closest to the focal sequence in the phylogenetic tree. In most cases these sequences were indeed attributed to subtype B with this phylogenetic method.

#### Phylogenetic inference

Maximum likelihood phylogenies were inferred using the software ExaML [59]. We used the GTR model of substitution with gamma-distributed rate heterogeneity among sites. We inferred the phylogeny separately on 2 sets of sequences: the full set (*N* = 2,028) and the subset of subtype B sequences (*N* = 1,581). We rooted the phylogenies using an outgroup (20 group N, O, P, and chimpanzee SIV references for the full set and 10 subtype D references for the subset of subtype B). For the 2 sets, we inferred 10 maximum likelihood phylogenies starting from 10 different starting points and retained the phylogeny with maximum likelihood among them. We additionally ran 100 bootstrapped phylogenies for each set.

### Measuring heritability using phylogenetic comparative methods

In order to measure heritability of SPVL, we fitted a series of stochastic models that describe how viral load evolves along the branch of the tree. Under all these models, the distribution of SPVL at the tip of the trees is a random drawing in a multivariate normal distribution whose mean vector and variance–covariance matrix, denoted (***μ***, **Σ**), depend on the model and its parameters and on the structure of the tree.

#### Brownian motion model

The BM model of evolution is classically used to explain similarity among species because of shared ancestry. It is behind phylogenetic mixed models [24,25] and Pagel’s lambda [26] and has recently been used to infer the heritability of SPVL in HIV [29]. The model assumes the character evolves according to Brownian motion along the phylogeny, starting from the ancestral character *g*_*a*_. The BM is characterized by its stochastic variance *σ*^2^. The resulting characters at the *n* tips of the phylogeny are a realization of a draw in a multivariate normal distribution with mean vector

$$\mu =g_{a}X$$

(here, ***X*** is the vector of 1 of length *n*) and covariance matrix with elements

$$\Sigma_{i,j}=\sigma^{2}t_{i,j}\text{ when }i\neq j$$

$$\Sigma_{i,j}=\sigma^{2}t_{i,j}+\epsilon^{2}\text{ when }i=j$$

where *t*_*i*,*j*_ is the phylogenetic distance between the ancestor of the whole tree and the most recent common ancestor of tips *i* and *j*, and *ϵ*^2^ quantifies all sources of variation which are not structured along the phylogeny, including measurement error and environmental factors. The likelihood is then given by the probability density function of the multivariate normal distribution, that is:

$$-2\text{log}\left(L\right)=n\text{log}\left(2\pi \right)+\text{log}\left(\left|\Sigma \right|\right)+\left(Y-g_{a}X\right)\prime \Sigma^{-1}\left(Y-g_{a}X\right)$$

where |.| denotes the determinant,.*'* the matrix transpose, and.^−1^ the matrix inverse.

For each value of (*σ*^2^, *ϵ*^2^) the parameter *g*_*a*_ could be directly replaced by its least square estimate

$$\hat{g_{a}}={\left(X^{\prime }\Sigma^{-1}X\right)}^{-1}\left(X^{\prime }\Sigma^{-1}Y\right)$$

Therefore, it was sufficient to optimize the likelihood with respect to (*σ*^2^, *ϵ*^2^) only. We did so using the Nelder–Mead method.

#### OU model

The OU model is similar to the BM model except that an additional force, which can be interpreted as stabilising selection, brings the character back to an optimum value *θ* [36]. The expected value of the infinitesimal change in character is E[*dy*] = −*α*(*y* − *θ*)*dt*, and the stochastic variance is V[*dy*] = *σ*^2^*dt* as in the BM model. In this model, the distribution of the character at the tips is also given by a multivariate normal distribution. The mean vector has elements

$$\mu_{i}=\left(1-{\text{e}}^{-\alpha t_{i}}\right)\theta +{\text{e}}^{-\alpha t_{i}}g_{a}$$

where *t*_*i*_ is the root to tip distance for tip *i*, and the covariance matrix has elements

$$\Sigma_{i,j}=\frac{\sigma^{2}}{2\alpha }\left(1-{\text{e}}^{-2\alpha t_{i,j}}\right)e^{-\alpha d_{i,j}}\text{ when }i\neq j$$

$$\Sigma_{i,j}=\frac{\sigma^{2}}{2\alpha }\left(1-{\text{e}}^{-2\alpha t_{i,j}}\right)e^{-\alpha d_{i,j}}+\epsilon^{2}\text{ when }i=j$$

where *d*_*i*,*j*_ is the patristic distance between tips *i* and *j* [36] (equal to 0 when *i = j*). We recover the expressions for the BM model in the limit when *α* → 0. In the limit where both *α* and *σ* are large, the covariance elements become 0 for off-diagonal elements and σ^2^/2*α* + *ϵ*^2^ for diagonal elements. In other words, the OU model converges to the null model, with the important difference that character variability will be interpreted as genetic instead of environmental. The expected value of the character, starting from *g*_*a*_ (when *t*_*i*_, the root-to-tip distance, is 0), converges to the optimum *θ* with a relaxation time inversely proportional to the strength of selection *α*. Selection tends to erase the covariance between the characters in 2 tips, which tends to 0 as the patristic distance between the tips increases.

In the OU model, the likelihood is

$$-2\text{log}\left(L\right)=n\text{log}\left(2\pi \right)+\text{log}\left(\left|\Sigma \right|\right)+{\left(Y-X\;\beta \right)}^{\prime }\Sigma^{-1}\left(Y-X\;\beta \right)$$

where ***X*** is now a matrix of dimension(*n*, 2), whose *i*th row is {$1-{\text{e}}^{-\alpha t_{i}},\;{\text{e}}^{-\alpha t_{i}}$}, which specifies the relationship between the expected character value and the coefficients, in our case the optimum and the ancestral character ***β*** = {*θ*, *g*_*a*_}′. As for Brownian motion, we can calculate the maximum likelihood estimate of {*θ*, *g*_*a*_} for given values of *α*, *σ*^2^, *ϵ*^2^

$$\beta ={\left(X^{\prime }\Sigma^{-1}X\right)}^{-1}\left(X^{\prime }\Sigma^{-1}Y\right)$$

then plug these ML estimates in the likelihood function and optimize on *α*, *σ*^2^, and *ϵ*^2^ only.

In practice, when optimizing the likelihood, we set an upper limit *α* = 10 to avoid convergence problems when both *α* and σ^2^ are large (in that limit, the 2 parameters are not identifiable).

#### Adding epidemiological covariates to the regression

Epidemiological covariates affecting viral load, such as sex, transmission mode, and age can be incorporated into the phylogenetic model by adding other columns in the matrix ***X***. Specifically, these extra columns will be the design matrix corresponding to the covariates of interest. Inference will then proceed as described above, and the maximum likelihood estimates of the phylogenetic parameters $\hat{\theta },\hat{g_{a}}$ and the effect of covariates will be obtained by the same formula, $\beta ={\left(X^{\prime }\Sigma^{-1}X\right)}^{-1}\left(X^{\prime }\Sigma^{-1}Y\right)$.

Computing the likelihood by inverting the **Σ** matrix would be computationally intensive for large datasets, but faster methods exist that directly compute the products of interest for the likelihood. We tested 3 implementations of the likelihood, which gave exactly the same result but differ in their details and performance: (i) a slow custom code computing matrix inversion using Cholesky decomposition, (ii) an adaptation of a linear-time algorithm developed by Ho and Ané [60], and (iii) a linear-time algorithm recently developed by Mitov and Stadler [35]. We used method (ii) for its speed and easy incorporation of covariates in the linear model.

#### Computing confidence intervals

We quantified uncertainty on the parameters and on heritability using parametric bootstrapping on the bootstrapped trees. This method combines uncertainty because of finite sample size and uncertainty in the phylogenetic tree inference. Confidence intervals represent the range of possible inferred parameters across samples if the true parameters are the ML parameters. For each of the 100 bootstrap trees, we simulated a stochastic outcome of the ML model and reinferred ML parameters from these simulations. The confidence intervals are the 2.5% and 97.5% percentiles of the resulting distribution.

To assess the significance of differences in heritability across the genome, we computed confidence intervals reflecting only the uncertainty in phylogenetic tree inference. The heritability estimates across the genome do not represent independent samples of the population but rather the same sample with different phylogenetic relationships. For each of the 100 bootstrap trees, we reinferred ML parameters from the data using the bootstrap tree instead of the maximum likelihood tree. The confidence intervals are the 2.5% and 97.5% percentiles of the resulting distribution.

#### Calculating heritability

Heritability of a trait is the fraction of the phenotypic variance in a population explained by genetic factors. Heritability is defined relative to a population and may change through time and in space. Given a subset of tips *S* ⊆ {1, …, *n*}, the traits at these tips are a realization of a random drawing in a multivariate normal (***μ***_*S*_, **Σ**_*S*_) where the subscript *S* indicates selection of elements of the subset *S* in the vector and matrix (***μ***, **Σ**), the overall mean and variance for the full set of tips. In all models above, the environmental component of the phenotype affects all traits independently, such that the covariance matrix can be rewritten **Σ = *Γ*** + ***I***_***n***_***ϵ***^**2**^, where ***Γ*** is the component of covariance attributable to shared ancestry and evolution along the branches. Moreover, the vector of means can be rewritten as *μ* = *γ* + *c*, where ***γ*** corresponds to the evolutionary process (a constant equal to the ancestral trait *g*_*a*_ in the BM model and not constant in the OU process) and ***c*** to epidemiological covariates. Thus, the vector of phenotypes ***Y***_***S***_ can be rewritten as

$$Y_{S}=c_{S}+G_{S}+{Ε}_{S}$$

where ***c*** are the predicted values corresponding to the epidemiological covariates, ***G***_*S*_ is the vector of genetic values drawn from $N\left(\gamma_{S},\Gamma_{S}\right)$, and ***E***_*S*_ is the vector of environmental values drawn from $N\left(0,I\;\epsilon^{2}\right)$. The phenotypic variance is the variance of elements in this vector. If the size of the subset is sufficiently large that we can neglect covariances between environmental effects and other factors, the phenotypic variance (the sample variance of the trait in the population) is

$$\text{V}\left[Y_{S}\right]=\text{V}\left[c_{S}\right]+\text{V}\left[G_{S}\right]+\text{V}\left[{Ε}_{S}\right]+2\text{cov}\left[c_{S},G_{S}\right]$$

In general, the phenotypic variance cannot be partitioned into a sum of components corresponding to genetic variance, variance because of epidemiological covariates, and environmental variance. This is because of the fact that the phenotypic variance is affected by interactions between genetic values and epidemiological covariates, as quantified by cov[***c***_*S*_, ***G***_*S*_]. Assuming large size of the subset, this term will be nonzero under the OU model when epidemiological covariates are included (i.e., the case in which the *g*_*i*_ are not all the same in expectation and the *c*_*i*_ are non-null).

Heritability can be defined as the ratio of the genetic variance over the phenotypic variance,

$$h_{\text{S}}^{2}=\frac{\text{V}\left[G_{S}\right]}{\text{V}\left[Y_{S}\right]}$$

Heritability is a distributed quantity, and will not exactly be the same across different realisations of the stochastic evolutionary process. Using simulation of the process, we can identify the part of the simulated character contributed by genetic factors ***G***_*S*_ and compute *h*^2^. We report results on the mean heritability computed over 1,000 stochastic simulations (Tables 1 and 2, S2 Table).

We next compute an analytical approximation for heritability as a function of model parameters. The full distribution of the phenotypic variance across realizations of the model would be hard to specify, but the expected value can be computed as

$$\text{E}\left[\text{V}\left[Y_{S}\right]\right]=\text{V}\left[c_{S}\right]+\text{E}\left[\text{V}\left[G_{S}\right]\right]+\text{E}\left[\text{V}\left[{Ε}_{S}\right]\right]+2\text{E}\left[\text{cov}\left[c_{S},G_{S}\right]\right]$$

$$\text{V}\left[c_{S}\right]=\frac{1}{s}\underset{i\in S}{\sum }c_{i}^{2}-\frac{1}{s^{2}}\underset{i\in S}{\sum }\underset{j\in S}{\sum }c_{i}c_{j}$$

$$\text{E}\left[\text{V}\left[G_{S}\right]\right]=\frac{1}{s}\underset{i\in S}{\sum }\left(\gamma_{i}^{2}+\Gamma_{i,i}\right)-\frac{1}{s^{2}}\underset{i\in S}{\sum }\underset{j\in S}{\sum }\left(\gamma_{i}\gamma_{j}+\Gamma_{i,j}\right)$$

$$\text{E}\left[\text{V}\left[{Ε}_{S}\right]\right]=\left(1-\frac{1}{s}\right)\epsilon^{2}$$

$$\text{E}\left[\text{cov}\left[c_{S},G_{S}\right]\right]=\frac{1}{s}\underset{i\in S}{\sum^{\text{​}}}\gamma_{i}c_{i}-\frac{1}{s^{2}}\sum_{i\in S}\underset{j\in S}{\sum^{\text{​}}}\gamma_{i}c_{j}$$

where *s* is the number of elements in subset *S*, *s* = |*S*|. Approximating the expected value of this ratio as the ratio of expected values, and neglecting the covariance between epidemiological covariates and genetic values, the expected heritability is

$$\text{E}\left[h_{S}^{2}\right]\approx \frac{\text{E}\left[\text{V}\left[G_{S}\right]\right]}{\text{E}\left[\text{V}\left[Y_{S}\right]\right]}=\frac{\text{E}\left[\text{V}\left[G_{S}\right]\right]}{\text{V}\left[c_{S}\right]+\text{E}\left[\text{V}\left[G_{S}\right]\right]+\text{E}\left[\text{V}\left[{Ε}_{S}\right]\right]}$$

This analytical expression for mean heritability was very accurate, as shown by systematic comparison with the mean heritability obtained across 1,000 simulations. The covariance between epidemiological covariates and genetic values, E[cov[***c***_***S***_, ***G***_*S*_]], was always very small in our dataset.

## Supporting information

S1 Fig Maximum likelihood subtype B phylogeny. The maximum likelihood phylogeny of viral sequences of subtype B used for the estimation of heritability (N = 1581), with edges coloured by viral load value (in log_10_ copies/mL). Black points show nodes with bootstrap values greater than 90%. The data used in the figure is provided as supplementary information.
(EPS)

S2 Fig Estimated heritability as a function of sample size. Estimated heritability under the BM (top panel) and OU (bottom panel) models, as a function of sample size. We randomly sampled 40 subsets of the full dataset (subtype B only, N = 1581) and inferred maximum likelihood parameters and heritability in each subset. Bullets show the maximum likelihood heritability and gray lines show the bootstrap confidence intervals. The dashed line represents the maximum likelihood estimate for the full dataset (N = 1581, point on the right). The data used in the figure is provided as supplementary information.
(EPS)

S3 Fig Increase in phenotypic variance in the data and predicted increase in genetic variance in the phylogenetic models. Bullets show the variance in GSVL (black) and SPVL (gray) among subtype B samples as a function of the time of the sample, calculated over 2-years intervals. The corresponding lines show the linear regression, with a significant increase for GSVL but not for SPVL when adjusting for covariates (S1 Table). Dotted lines show the predicted increase in genetic variance under the maximum likelihood OU model. Dashed lines show the predicted increase in genetic variance under the maximum likelihood BM model. These predictions were computed by simulating the maximum likelihood model, and calculating the mean genetic variance over 1000 realisations of the process and for each set of tips (corresponding to patients sampled in 1985–1986, 1987–1988, etc). The data used in the figure is provided in S1 Data.
(EPS)

S4 Fig Linkage disequilibrium in the population as a function of the distance separating pairs of loci. We considered all subtype B sequences (N = 1581), and calculated the linkage disequilibrium for 100,000 pairs of positions where the two most common nucleotides have frequency greater than 0.01. Linkage disequilibrium was calculated as D = (X_AB_-p_A_ p_B_)/√[p_A_ (1-p_A_) p_B_ (1-p_B_)] where A denotes the most common allele (nucleotide) at the first position, B denotes the most common allele at the second position, X_AB_ is the frequency of the genotype AB and p_A_ and p_B_ the frequencies of alleles A and B (ignoring other nucleotides present at smaller frequencies at the locus). Positive linkage indicates association between the two most common alleles. We show average linkage disequilibrium as function of the distance between positions. Weak positive linkage even at long distances may be due to shared ancestry. The data used in the figure is provided in S1 Data.
(EPS)

S5 Fig Schematic of a donor-recipient pair. On the left, the genealogy of a donor transmitting to a recipient. Arrows denote the time of sampling and measurement of each partner. We assume the donor is measured and sampled before the branching in the genealogy (which may be anterior to the transmission event because of within-host diversity). On the right, the resulting phylogeny. We assume the donor trait is equal to the trait of the MRCA of the donor and the recipient.
(EPS)

S1 Table Analysis of temporal trends in GSVL and SPVL. (DOCX)

S2 Table Analysis of heritability stratified by country, gender, mode of transmission. (DOCX)

S3 Table Analysis of heritability for another viral load measure, for a linear model with country included as a covariate, and for other inclusion criteria for viral sequences. (DOCX)

S4 Table Analysis of variance for three viral load measures, for the subset of patients infected by subtype B virus. (DOCX)

S5 Table Analysis of variance for GSVL and SPVL viral load measures, for patients infected by all subtypes (N = 2028). (DOCX)

S1 Text The relationship between SPVL and GSVL. (DOCX)

S2 Text The relationship between phylogenetic heritability and donor-recipient regression. (DOCX)

S3 Text Cohort members. (DOCX)

S1 Data Data for Figs 1B, 1C, 2, S2, S3 and S4. (ZIP)

## Abbreviations

AIC: Akaike Information Criterion
BM: Brownian motion
CTL: cytotoxic T lymphocyte
GSVL: gold standard viral load
IDU: injecting drug user
ML: maximum likelihood
MSM: men having sex with men
OU: Ornstein–Uhlenbeck
SPVL: set-point viral load
