# Correction: Viral genetic variation accounts for a third of variability in HIV-1 set-point viral load in Europe

**DOI:** 10.1371/journal.pbio.1002608

**Published:** 2017-07-13

**Authors:** François Blanquart, Chris Wymant, Marion Cornelissen, Astrid Gall, Margreet Bakker, Daniela Bezemer, Matthew Hall, Mariska Hillebregt, Swee Hoe Ong, Jan Albert, Norbert Bannert, Jacques Fellay, Katrien Fransen, Annabelle J. Gourlay, M. Kate Grabowski, Barbara Gunsenheimer-Bartmeyer, Huldrych F. Günthard, Pia Kivelä, Roger Kouyos, Oliver Laeyendecker, Kirsi Liitsola, Laurence Meyer, Kholoud Porter, Matti Ristola, Ard van Sighem, Guido Vanham, Ben Berkhout, Paul Kellam, Peter Reiss, Christophe Fraser, BEEHIVE collaboration

The affiliation for the 25th author is incorrect. Matti Ristola is not affiliated with #19 but with #17 Department of Infectious Diseases, Helsinki University Hospital, Helsinki, Finland. Please see the full, correct byline and affiliations below.

François Blanquart^1^*, Chris Wymant^1,2^, Marion Cornelissen^3^, Astrid Gall^4¤^, Margreet Bakker^3^, Daniela Bezemer^5^, Matthew Hall^2^, Mariska Hillebregt^5^, Swee Hoe Ong^4^, Jan Albert^6,7^, Norbert Bannert^8^, Jacques Fellay^9,10^, Katrien Fransen^11^, Annabelle J. Gourlay^12^, M. Kate Grabowski^13^, Barbara Gunsenheimer-Bartmeyer^14^, Huldrych F. Günthard^15,16^, Pia Kivelä ^17^, Roger Kouyos^15,16^, Oliver Laeyendecker^18^, Kirsi Liitsola^19^, Laurence Meyer^20^, Kholoud Porter^12^, Matti Ristola^17^, Ard van Sighem^5^, Guido Vanham^21^, Ben Berkhout^3^, Paul Kellam^22,23^, Peter Reiss^5,24^, Christophe Fraser ^1,2*^, BEEHIVE collaboration^¶^

**1** Department of Infectious Disease Epidemiology, Imperial College London, London, United Kingdom, **2** Big Data Institute, Li Ka Shing Centre for Health Information and Discovery, Nuffield Department of Medicine, University of Oxford, Oxford, United Kingdom, **3** Laboratory of Experimental Virology, Department of Medical Microbiology, Center for Infection and Immunity Amsterdam (CINIMA), Academic Medical Center of the University of Amsterdam, Amsterdam, the Netherlands, **4** Wellcome Trust Sanger Institute, Wellcome Genome Campus, Hinxton, Cambridge, United Kingdom, **5** Stichting HIV Monitoring, Amsterdam, the Netherlands, **6** Department of Microbiology, Tumor and Cell Biology, Karolinska Institutet, Stockholm, Sweden, **7** Department of Clinical Microbiology, Karolinska University Hospital, Stockholm, Sweden, **8** Division for HIV and other Retroviruses, Robert Koch Institute, Berlin, Germany, **9** School of Life Sciences, Ecole Polytechnique Fédérale de Lausanne, Lausanne, Switzerland, **10** Swiss Institute of Bioinformatics, Lausanne, Switzerland, **11** HIV/STI reference laboratory, WHO collaborating centre, Institute of Tropical Medicine, Department of Clinical Science, Antwerpen, Belgium, **12** Department of Infection and Population Health, University College London, London, United Kingdom, **13** Department of Epidemiology, John Hopkins University, Baltimore, Maryland, United States of America, **14** Department of Infectious Disease Epidemiology, Robert Koch-Institute, Berlin, Germany, **15** Division of Infectious Diseases and Hospital Epidemiology, University Hospital Zurich, Zurich, Switzerland, **16** Institute of Medical Virology, University of Zurich, Zurich, Switzerland, **17** Department of Infectious Diseases, Helsinki University Hospital, Helsinki, Finland, **18** Laboratory of Immunoregulation, National Institute of Allergy and Infectious Diseases, National Institutes of Health, Baltimore, Maryland, United States of America, **19** Department of Health Security, National Institute for Health and Welfare, Helsinki, Finland, **20** INSERM CESP U1018, Université Paris Sud, Université Paris Saclay, APHP, Service de Santé Publique, Hôpital de Bicêtre, Le Kremlin-Bicêtre, France, **21** Virology Unit, Immunovirology Research Pole, Biomedical Sciences Department, Institute of Tropical Medicine, Antwerpen, Belgium, **22** Kymab Ltd, Cambridge, United Kingdom, **23** Division of Infectious Diseases, Department of Medicine, Imperial College London, London, United Kingdom, **24** Department of Global Health, Academic Medical Center, Amsterdam, the Netherlands

¤ Current address: Department of Veterinary Medicine, University of Cambridge, Cambridge, United Kingdom

¶ Membership of the BEEHIVE collaboration is provided in the Acknowledgments.

* f.blanquart@imperial.ac.uk (FB); christophe.fraser@bdi.ox.ac.uk (CF)
