# Supplementary material for: Viral genetic variation accounts for a third of variability in HIV-1 set-point viral load in Europe
Source: PLoS Biol. 2017 Jun 12;15(6):e2001855. doi: 10.1371/journal.pbio.2001855 (PMC5467800; doi:10.1371/journal.pbio.2001855)
Supplement: S1 Table — (DOCX) [file pbio.2001855.s006.docx]

### Supplementary Table 1: analysis of temporal trends in GSVL and SPVL

For both GSVL and SPVL measures, we fitted a linear model includes a constant, a fixed effect of calendar time, and the fixed effects of the covariates gender, transmission mode, age, ethnicity, and assay. The variance is a constant (“baseline variance”) plus an effect of calendar time (“calendar time-variance”) describing how variance changes over time. We report p-values for the “calendar time” fixed effect (describing the evolution of average viral load over calendar time) and for the effect of calendar time on the variance (“p-value variance”).

| **country** | **measure** | **N** | **intercept** | **calendar time** | **baseline variance** | **calendar time- variance** | **p-value calendar time** | **p-value variance** |
| --- | --- | --- | --- | --- | --- | --- | --- | --- |
| BE | GSVL | 40 | 5.31 | -0.027 | 1.88 | -0.052 | 0.735 | 0.126 |
| CH | GSVL | 742 | 4.41 | -0.015 | 0.4 | 0.003 | 0.012 | 0.554 |
| FR | GSVL | 278 | 4.09 | 0.011 | 0.64 | -0.007 | 0.452 | 0.667 |
| NL | GSVL | 434 | 4.3 | 0.015 | 0.28 | 0.01 | 0.007 | 0.006 |
| UK | GSVL | 87 | 4.6 | -0.021 | 0.12 | 0.019 | 0.376 | 0.508 |
| **all** | GSVL | 1581 | 4.6 | -0.006 | 0.31 | 0.01 | 0.058 | 0.002 |
| **all** | SPVL | 1581 | 4.06 | 0.002 | 0.46 | 0.001 | 0.636 | 0.786 |
