# Supplementary material for: Viral genetic variation accounts for a third of variability in HIV-1 set-point viral load in Europe
Source: PLoS Biol. 2017 Jun 12;15(6):e2001855. doi: 10.1371/journal.pbio.2001855 (PMC5467800; doi:10.1371/journal.pbio.2001855)
Supplement: S2 Table — (DOCX) [file pbio.2001855.s007.docx]

**Supplementary Table 2: Analysis of heritability stratified by country, gender, mode of transmission**

Summary of model fit for GSVL, for subtype B, stratified by country (BE, CH, FR, NL, UK), gender (Male and Female), and mode of transmission (HET, IDU, MSM). There was statistical support for a contribution of viral genetic factors to the viral load only in The Netherlands, in the subset of males, and in the subset of MSM.

| **subset** | **model** | **N** | **AIC** | **AIC weight** | **V_E_** | **σ^2^** | **α** | **optimum θ** | **h^2^** |
| --- | --- | --- | --- | --- | --- | --- | --- | --- | --- |
| BE | NULL | 40 | 102 | 0.67 | 0.7 [0.31; 1.1] | - | - | - | 0 [0; 0] |
| BE | BM | 40 | 104 | 0.24 | 0.68 [0.081; 0.98] | 1.1e-06 [1e-06; 3] | - | - | 0 [0; 0.87] |
| BE | OU | 40 | 106 | 0.09 | 0.65 [1e-05; 0.75] | 1.2e-06 [1e-05; 10] | 10 [1e-05; 10] | 5.3 [-41000; 980000] | 0.05 [0; 1] |
| CH | NULL | 742 | 1571 | 0.58 | 0.48 [0.43; 0.53] | - | - | - | 0 [0; 0] |
| CH | BM | 742 | 1573 | 0.21 | 0.47 [0.43; 0.51] | 1e-06 [1e-06; 0.14] | - | - | 0 [0; 0.06] |
| CH | OU | 742 | 1573.1 | 0.2 | 0.36 [0.29; 0.47] | 2.3 [1e-05; 3.7] | 10 [1.7; 10] | 4 [3.6; 5] | 0.23 [0; 0.39] |
| FR | NULL | 278 | 602.4 | 0.66 | 0.5 [0.42; 0.59] | - | - | - | 0 [0; 0] |
| FR | BM | 278 | 604.4 | 0.24 | 0.49 [0.39; 0.56] | 1e-06 [1e-06; 0.28] | - | - | 0 [0; 0.09] |
| FR | OU | 278 | 606.2 | 0.1 | 0.49 [0.31; 0.54] | 1e-06 [1e-05; 3.4] | 10 [1.2; 10] | 4.6 [3.9; 6.5] | 0.01 [0; 0.34] |
| NL | NULL | 434 | 918.7 | 0 | 0.47 [0.4; 0.53] | - | - | - | 0 [0; 0] |
| NL | BM | 434 | 904.9 | 0.69 | 0.35 [0.27; 0.42] | 0.68 [0.18; 1.1] | - | - | 0.25 [0.08; 0.39] |
| NL | OU | 434 | 906.5 | 0.31 | 0.34 [0.18; 0.4] | 0.89 [0.59; 5.8] | 1.6 [0.54; 10] | 5.4 [4.3; 6.8] | 0.26 [0.12; 0.6] |
| UK | NULL | 87 | 200.2 | 0.22 | 0.56 [0.42; 0.68] | - | - | - | 0 [0; 0] |
| UK | BM | 87 | 198.3 | 0.58 | 0.27 [0.035; 0.58] | 1.3 [1e-06; 2.8] | - | - | 0.49 [0; 0.93] |
| UK | OU | 87 | 200.5 | 0.19 | 0.018 [1e-05; 0.56] | 10 [1e-05; 10] | 10 [1e-05; 10] | 3.9 [-230000; 78000] | 0.96 [0; 1] |
| Male | NULL | 1446 | 3131 | 0 | 0.51 [0.46; 0.54] | - | - | - | 0 [0; 0] |
| Male | BM | 1446 | 3115.6 | 0.35 | 0.43 [0.38; 0.46] | 0.44 [0.17; 0.69] | - | - | 0.16 [0.07; 0.23] |
| Male | OU | 1446 | 3114.4 | 0.65 | 0.34 [0.27; 0.42] | 3.4 [0.61; 4.6] | 10 [2.5; 10] | 4.5 [3.6; 5.5] | 0.32 [0.14; 0.46] |
| Female | NULL | 135 | 321.1 | 0.66 | 0.6 [0.46; 0.72] | - | - | - | 0 [0; 0] |
| Female | BM | 135 | 323.1 | 0.24 | 0.57 [0.35; 0.72] | 1e-06 [1e-06; 0.73] | - | - | 0 [0; 0.23] |
| Female | OU | 135 | 325 | 0.09 | 0.56 [0.019; 0.63] | 4.6e-06 [1e-05; 10] | 9.6 [1e-05; 10] | 4.6 [-370000; 22000] | 0.01 [0; 0.91] |
| HET | NULL | 211 | 493.2 | 0.68 | 0.58 [0.45; 0.67] | - | - | - | 0 [0; 0] |
| HET | BM | 211 | 495.2 | 0.25 | 0.55 [0.42; 0.65] | 1e-06 [1e-06; 0.41] | - | - | 0 [0; 0.13] |
| HET | OU | 211 | 497.9 | 0.07 | 0.25 [1e-05; 0.61] | 6.4 [1e-05; 10] | 10 [1e-05; 10] | 4.7 [-280000; 8900] | 0.51 [0; 0.97] |
| IDU | NULL | 110 | 240.8 | 0.69 | 0.5 [0.34; 0.6] | - | - | - | 0 [0; 0] |
| IDU | BM | 110 | 242.8 | 0.25 | 0.49 [0.38; 0.59] | 1e-06 [1e-06; 0.19] | - | - | 0 [0; 0.09] |
| IDU | OU | 110 | 245.7 | 0.06 | 0.089 [1e-05; 0.44] | 9.8 [1e-05; 10] | 10 [1.8e-05; 10] | 4 [-150000; 4.9] | 0.84 [0.08; 0.99] |
| MSM | NULL | 1196 | 2595.8 | 0 | 0.51 [0.46; 0.55] | - | - | - | 0 [0; 0] |
| MSM | BM | 1196 | 2580.8 | 0.56 | 0.42 [0.37; 0.46] | 0.47 [0.22; 0.71] | - | - | 0.17 [0.08; 0.26] |
| MSM | OU | 1196 | 2581.3 | 0.44 | 0.35 [0.28; 0.45] | 3.2 [0.31; 4.9] | 10 [1.2; 10] | 4.4 [4; 5.1] | 0.3 [0.08; 0.44] |

Note that on small cohorts, and when the OU model is not well supported, the inference on bootstrapped pseudo-datasets (used to generate confidence intervals) may converge to solutions where stabilising selection α is extremely small and the inferred optimum may take on very small or large values.
