# Supplementary material for: Viral genetic variation accounts for a third of variability in HIV-1 set-point viral load in Europe
Source: PLoS Biol. 2017 Jun 12;15(6):e2001855. doi: 10.1371/journal.pbio.2001855 (PMC5467800; doi:10.1371/journal.pbio.2001855)
Supplement: S3 Table — (DOCX) [file pbio.2001855.s008.docx]

**Supplementary Table 3: Analysis of heritability for another viral load measure, for a linear model with country included as a covariate, and for other inclusion criteria for viral sequences.** The first three lines (condition “main”, measure “single VL”) present inference for an alternative viral load measure, a single viral load among those included in SPVL, measured at the date closest to the date of the sample used for viral sequencing. The following lines (condition “with country”) present inference for GSVL, when the covariate “country” was included in the phylogenetic regression. The remaining lines test robustness of the analysis to the details of the processing of the viral sequences before phylogenetic analysis, for GSVL. In condition “with DRM/CTL”, the drug resistance mutations and CTL escape sites were *not* removed before phylogenetic analysis. In condition “lower cov.”, sequence was included when supported by a coverage of at least 15 reads (for MiSeq technology) or 150 reads (for HiSeq technology) (instead of 30 and 300 respectively for the main analysis). In condition “lim 1000 bp”, only sequences greater than 1000 bp were included for phylogenetic analysis (instead of > 500 bp for the main analysis).

| **condition** | **measure** | **model** | **N** | **AIC** | **AIC weight** | **V_E_** | **σ^2^** | **α** | **optimum θ** | **h^2^** |
| --- | --- | --- | --- | --- | --- | --- | --- | --- | --- | --- |
| main | single VL | NULL | 1581 | 3412.5 | 0 | 0.5 [0.47; 0.53] | - | - | - | 0 [0; 0] |
| main | single VL | BM | 1581 | 3396.7 | 0.24 | 0.42 [0.38; 0.46] | 0.42 [0.18; 0.65] | - | - | 0.14 [0.07; 0.22] |
| main | single VL | OU | 1581 | 3394.4 | 0.76 | 0.34 [0.28; 0.42] | 3.3 [1.2; 4.3] | 10 [4.4; 10] | 4.4 [3.8; 5.1] | 0.3 [0.14; 0.39] |
| with country | GSVL | NULL | 1581 | 3386.8 | 0 | 0.49 [0.45; 0.53] | - | - | - | 0 [0; 0] |
| with country | GSVL | BM | 1581 | 3377.8 | 0.26 | 0.43 [0.38; 0.46] | 0.35 [0.13; 0.59] | - | - | 0.12 [0.04; 0.22] |
| with country | GSVL | OU | 1581 | 3375.7 | 0.74 | 0.35 [0.28; 0.44] | 2.8 [0.51; 3.9] | 10 [3.4; 10] | 4.7 [3.6; 5.5] | 0.25 [0.09; 0.39] |
| with DRM/CTL | GSVL | NULL | 1581 | 3461.7 | 0 | 0.52 [0.48; 0.55] | - | - | - | 0 |
| with DRM/CTL | GSVL | BM | 1581 | 3445.6 | 0.04 | 0.44 | 0.42 | - | - | 0.14 |
| with DRM/CTL | GSVL | OU | 1581 | 3439.2 | 0.96 | 0.33 | 3.8 | 10 | 4.3 | 0.34 |
| lower cov. | GSVL | NULL | 1583 | 3476.8 | 0 | 0.52 [0.48; 0.55] | - | - | - | 0 |
| lower cov. | GSVL | BM | 1583 | 3458.2 | 0.32 | 0.43 | 0.52 | - | - | 0.17 |
| lower cov. | GSVL | OU | 1583 | 3456.7 | 0.68 | 0.35 | 3.4 | 9.5 | 4.4 | 0.32 |
| lim 1000 bp | GSVL | NULL | 1576 | 3425.4 | 0 | 0.51 [0.47; 0.54] | - | - | - | 0 |
| lim 1000 bp | GSVL | BM | 1576 | 3401.5 | 0.34 | 0.41 | 0.54 | - | - | 0.19 |
| lim 1000 bp | GSVL | OU | 1576 | 3400.2 | 0.66 | 0.35 | 2.4 | 6.7 | 4.3 | 0.3 |
