# Supplementary material for: Viral genetic variation accounts for a third of variability in HIV-1 set-point viral load in Europe
Source: PLoS Biol. 2017 Jun 12;15(6):e2001855. doi: 10.1371/journal.pbio.2001855 (PMC5467800; doi:10.1371/journal.pbio.2001855)
Supplement: S4 Table — (DOCX) [file pbio.2001855.s009.docx]

| **measure** | **factor** | **sum of squares** | **d.f.** | **F value** | **p value** |
| --- | --- | --- | --- | --- | --- |
| GSVL | gender | 7.2 | 1 | 13.9 | 2.00E-04 |
|  | transmission mode | 2.4 | 3 | 1.5 | 0.2 |
|  | age category | 4.1 | 4 | 2 | 0.099 |
|  | ethnicity | 3.1 | 2 | 3 | 0.052 |
|  | assay | 0.7 | 1 | 1.4 | 0.24 |
|  | residuals | 813.3 | 1569 | - | - |
| SPVL | gender | 7.6 | 1 | 15.7 | 7.90E-05 |
|  | transmission mode | 2 | 3 | 1.4 | 0.24 |
|  | age category | 3.3 | 4 | 1.7 | 0.14 |
|  | ethnicity | 0.5 | 2 | 0.5 | 0.61 |
|  | assay | 10.2 | 6 | 3.5 | 0.0019 |
|  | residuals | 755.7 | 1564 | - | - |
| Single VL | gender | 6.6 | 1 | 13.1 | 3.00E-04 |
|  | transmission mode | 1.7 | 3 | 1.1 | 0.34 |
|  | age category | 3.9 | 4 | 1.9 | 0.1 |
|  | ethnicity | 0.8 | 2 | 0.8 | 0.43 |
|  | assay | 21.8 | 6 | 7.3 | 1.10E-07 |
|  | residuals | 783.3 | 1564 | - | - |

**Supplementary Table 4: Analysis of variance for three viral load measures, for the subset of patients infected by subtype B virus.** These linear models do not include a phylogenetic effect (“null model”). Levels of significance were calculated using a type II analysis of variance.
