# Supplementary material for: Viral genetic variation accounts for a third of variability in HIV-1 set-point viral load in Europe
Source: PLoS Biol. 2017 Jun 12;15(6):e2001855. doi: 10.1371/journal.pbio.2001855 (PMC5467800; doi:10.1371/journal.pbio.2001855)
Supplement: S5 Table — (DOCX) [file pbio.2001855.s010.docx]

| **measure** | **factor** | **sum of squares** | **d.f.** | **F value** | **p value** |
| --- | --- | --- | --- | --- | --- |
| GSVL | gender | 11.1 | 1 | 21.1 | 4.70E-06 |
|  | transmission mode | 6.1 | 3 | 3.9 | 0.0089 |
|  | age category | 4.4 | 4 | 2.1 | 0.083 |
|  | ethnicity | 3.6 | 2 | 3.4 | 0.032 |
|  | assay | 1.4 | 1 | 2.7 | 0.1 |
|  | subtype | 2.2 | 6 | 0.7 | 0.65 |
|  | residuals | 1062.7 | 2010 | - | - |
| SPVL | gender | 11.5 | 1 | 22.6 | 2.20E-06 |
|  | transmission mode | 6 | 3 | 3.9 | 0.0083 |
|  | age category | 3.9 | 4 | 1.9 | 0.1 |
|  | ethnicity | 1.1 | 2 | 1.1 | 0.35 |
|  | assay | 8 | 6 | 2.6 | 0.016 |
|  | subtype | 1.7 | 6 | 0.5 | 0.77 |
|  | residuals | 1017.3 | 2005 | - | - |

**Supplementary Table 5: Analysis of variance for GSVL and SPVL viral load measures, for patients infected by all subtypes (N = 2028).** These linear models do not include a phylogenetic effect (“null model”). Subtype was included in the regression (in contrast to the main analysis). Levels of significance were calculated using a type II analysis of variance.
