# Supplementary material for: Viral genetic variation accounts for a third of variability in HIV-1 set-point viral load in Europe
Source: PLoS Biol. 2017 Jun 12;15(6):e2001855. doi: 10.1371/journal.pbio.2001855 (PMC5467800; doi:10.1371/journal.pbio.2001855)
Supplement: S1 Text — (DOCX) [file pbio.2001855.s011.docx]

### Supplementary text 1: The relationship between SPVL and GSVL

Heritability of GSVL versus heritability of SPVL

The method used to define SPVL determines both the estimate and the interpretation of heritability. If SPVL averages several viraemia measures, and the viral genotype is the consensus of the whole viral quasispecies in the patient, then SPVL is the property of the whole course of infection, and heritability is the fraction of variance in SPVL explained by the consensus genotype. However if viral load is measured at a single time point, and viral genotype is also measured at the same time-point, then heritability is the fraction of variance explained by these more specific genotypes sampled at these time-points. The former encompasses the effects of within-host selection and evolution, the latter is specific to it at some defined time-point in the course of infection.

Viral load was either GSVL, i.e. viraemia re-measured on the same sample as used for viral genetics, using a standardized choice of assay; or the traditional SPVL measure, which is classically defined as the mean of log_10_ viral load for samples from multiple longitudinal samples during a defined period after first HIV positive test. These two measures can be modeled as the sum of several effects:

$$gsvl=g+\gamma_{1}+e_{host}+\epsilon_{1}$$

$$spvl=g+\gamma_{2}+e_{host}+\epsilon_{2}$$

For comparison, we also inferred the parameters of the BM model for another viral load measure, defined as the viral load, among those used in SPVL, measured at the date closest to the date where the viral sequence was sampled. This measure can be modeled as:

$$single\_vl=g+\gamma_{3}+e_{host}+\epsilon_{3}$$

The three measures have in common a viral genetic effect (virulence) *g* that represents the contribution of the viral genetics that is common to the three measures. This is the contribution of a reference viral sequence, for example the founder virus of the infection, or a consensus viral sequence summarizing viral genetics over the population and over the time period at which viral loads were measured. The host effect, $e_{host}$, is the same in all three measures. The measurement error is called $\epsilon_{1}$ for GSVL, $\epsilon_{2}$ for SPVL and $\epsilon_{3}$ for the single viral load. Measurement error is generated by variability in assays and error variance, and is reduced by assay standardization (for GSVL) but also by including multiple measurements (for SPVL).

We found, as expected, that the assay contributed more to the variance of SPVL and single VL than to the variance of GSVL, confirming the better standardization of the new GSVL measure (S4 Table). Within the phylogenetic regression, the environmental variance, generated by the error variance and host variability (but not by assay variability, as the regression is adjusted for assay) was similar across the three measures at around 0.42 – 0.43 (Table 1, S3 Table). The multiple measurements included in SPVL do not reduce environmental variance compared to GSVL / single VL, suggesting temporal variability in host properties does not contribute much to variation in viral load.

Viral evolution within the host causes deviations from the reference sequence and this will affect viraemia. The corresponding effect is $\gamma_{1}$ for GSVL, representing the contribution of the consensus viral sequence at the time of sampling; $\gamma_{2}$ for SPVL, which represents the contributions of the consensus sequences at the multiple time points where the viral loads in SPVL were measured; and $\gamma_{3}$ for the single viral load, representing the contribution of the consensus viral sequence at the time of sampling. We chose the single viral load with date closest to the date when the sequence was sampled in order to minimize the difference between $\gamma_{3}$ and $\gamma_{1}$.

The parameter σ^2^ of the BM phylogenetic model quantifies how character evolution on the tree gives rise to the $g+\gamma_{1}$ (for GSVL), $g+\gamma_{2}$ (for SPVL) and $g+\gamma_{3}$ (for the single VL) components. Heritability is significantly different for GSVL versus SPVL if and only if the estimated σ^2^  are significantly different for the two measures. In our case, σ^2^ = 0.50 for GSVL, σ^2^ = 0.35 for SPVL (Table 1) and σ^2^ = 0.42 for single viral load. Confidence intervals for this parameter largely overlapped, but these CI take into account uncertainty on *g* and *e_host_*, which is probably too conservative because we know *g* and *e_host_* are the same across the three measures.

What may explain the higher genetic variance for GSVL? We hypothesise that the genetic variance estimated from the phylogenetic approach directly corresponds to the genetic value of GSVL, that is $g+\gamma_{1}$, because the sequence was obtained from the same sample where GSVL was measured. In contrast, both SPVL and single viral load measures, the sequence does not correspond exactly to the viral sequence determining the measured trait, and therefore variation due to the corresponding genetic differences $\gamma_{2}$ and $\gamma_{3}$ may be interpreted as environmental variation and not genetic variation. If this interpretation is correct, single VL has a higher genetic variance than SPVL because the single VL was chosen to minimize the difference with the viral sequence. Accordingly, the parameter σ^2^ of the BM model for single VL (σ^2^ = 0.42) is intermediate between that of SPVL (σ^2^ = 0.35) and that of GSVL (σ^2^ = 0.50), supporting this interpretation (although differences in parameters are small). Moreover, the inferred heritability under the best-fitting OU model was 30% for the single VL, close to the 31% of GSVL and higher than 21% for SPVL.
