# Supplementary material for: Viral genetic variation accounts for a third of variability in HIV-1 set-point viral load in Europe
Source: PLoS Biol. 2017 Jun 12;15(6):e2001855. doi: 10.1371/journal.pbio.2001855 (PMC5467800; doi:10.1371/journal.pbio.2001855)
Supplement: S2 Text — (DOCX) [file pbio.2001855.s012.docx]

### Supplementary text 2: The relationship between phylogenetic heritability and donor-recipient regression

Heritability, the fraction of phenotypic variance attributable to genetic variance, is also equal under some assumptions to the regression coefficient of the offspring’s traits against the parents’ traits [1]. By analogy, for pathogens, heritability may be estimated by regressing the recipient’s viral load onto the donor’s viral load in known transmission pairs [2]. Transmission pairs can be identified using epidemiological information (known partnerships), or by focusing on pairs of viral sequences that are genetically similar such that the two corresponding persons are more likely to be a transmission pair [3,4]. Here we investigate the link between heritability defined as the fraction of phenotypic variance attributable to genetic variance under a model of character evolution on a phylogeny (the definition we adopt here), and the donor-recipient regression coefficient.

Let us assume that we have identified a set of *N* donors and recipients in an HIV epidemic, that we have collected viral samples of these individuals and constructed a phylogeny, and measured the viral load of donors $\boldsymbol{y}_{D}$ and the viral load of recipients $\boldsymbol{y}_{R}$. Let us further assume that each donor was sequenced early enough (in the course of within-host evolution) that the resulting viral sequence is ancestral to the corresponding recipient’s sequence (S5 Fig). The time between the donor and the recipient measurements is assumed to be the same for all transmission pairs for simplicity, and equal to $T_{DR}$ in terms of phylogenetic distance (which is proportional to calendar time under the assumption of the molecular clock).

We show that (i) in the limit where $T_{DR}$ is small, the donor-recipient regression coefficient is equal to the fraction of phenotypic variance attributable to genetic variance whatever the model of character evolution; (ii) under BM, the donor-recipient regression coefficient is equal to the fraction of phenotypic variance attributable to genetic variance regardless of $T_{DR}$; (iii) under OU, the donor-recipient regression coefficient is equal to the fraction of phenotypic variance attributable to genetic variance when $T_{DR}=0$, then decreases as $T_{DR}$ increases (when the tree is ultrametric).

The regression coefficient between the characters of donors $\boldsymbol{y}_{D}$ and the characters of recipients $\boldsymbol{y}_{R}$ is:

$$\beta=\frac{cov[\boldsymbol{y}_{D},\boldsymbol{y}_{R}]}{V[\boldsymbol{y}_{D}]}=\frac{cov[\boldsymbol{g}_{D}+\boldsymbol{e}_{D},\boldsymbol{g}_{D}+\boldsymbol{e}_{R}+\boldsymbol{\delta}_{DR}]}{V[\boldsymbol{g}_{D}+\boldsymbol{e}_{D}]}$$

where $\boldsymbol{g}_{D}$ is the vector of genetic values of the donors and is drawn in $\mathcal{N}\left( \boldsymbol{\gamma},\boldsymbol{\Gamma} \right)$, $\boldsymbol{e}_{D}$ is the vector of environmental values of the donors and is drawn in $\mathcal{N}\left( \boldsymbol{0},\boldsymbol{E} \right)$. $\boldsymbol{\delta}_{DR}$ is the vector of change in viral load due to evolution that happened during the time interval $T_{DR}$ between the donor and the recipient. We derive results for the regression coefficient, but under the assumption that the variance of the character is the same in donor and recipient, these results will be valid for the correlation coefficient. Environmental components are assumed to be independent between the donor and the recipient, and independent of the genetic values and the change in viral load. We can thus neglect the covariances between the environmental components and all other terms if the sample size is large enough, and the regression coefficient simplifies to:

$$\beta\approx\frac{V\left[ \boldsymbol{g}_{D} \right]+cov\left[ \boldsymbol{g}_{D},\boldsymbol{\delta}_{DR} \right]}{V\left[ \boldsymbol{g}_{D} \right]+V[\boldsymbol{e}_{D}]}$$

where the variance $V\left[ . \right]$ and covariance $\mathrm{cov}\left[ .,. \right]$ are taken over the sample of *N* donor-recipient pairs. The distribution of the $\boldsymbol{\delta}_{DR}$ depends on the model of character evolution on the phylogeny (Brownian Motion or Ornstein-Uhlenbeck). However, in the limit where the donor virus was measured just before transmission, and the recipient virus was measured just after transmission ($T_{DR}$ is small), the two viruses have almost the same genetic value ($\boldsymbol{\delta}_{DR}$ is small) and therefore the regression coefficient will be equal to heritability regardless of the model of character evolution (result i).

Brownian Motion (BM) model of evolution:

Under BM, the vector of character change $\boldsymbol{\delta}_{DR}$ is drawn in $\mathcal{N}\left( \boldsymbol{0},\boldsymbol{I}T_{DR}\sigma^{2} \right)$ and is independent of the genetic values $\boldsymbol{g}_{D}$. Thus, if the number of pairs is large, the covariance can be neglected, and the regression coefficient is approximately:

$$\beta_{S}\approx\frac{V\left[ \boldsymbol{g}_{D} \right]}{V\left[ \boldsymbol{g}_{D} \right]+V[\boldsymbol{e}_{D}]}$$

Thus, heritability as estimated by donor-recipient regression will be identical to heritability as estimated by fraction of phenotypic variance explained by genetic variance. An important prediction of the Brownian motion model of evolution is that heritability does not depend on the time between the sample in the donor and recipient $T_{DR}$ (result ii). What matters for heritability is only how much common ancestry the donor and recipient viruses share, as quantified by the elements of the matrix $\boldsymbol{\Gamma}$.

Ornstein-Uhlenbeck (OU) model of evolution

Under OU, the vector of character change $\boldsymbol{\delta}_{DR}$ is drawn in

$$\mathcal{N}\left( \left( 1-e^{-\alpha T_{DR}} \right)\theta+\left( e^{-\alpha T_{DR}}-1 \right)\boldsymbol{g}_{D}, \boldsymbol{I}\frac{\sigma^{2}}{2\alpha}\left( 1-e^{-2\alpha T_{DR}} \right) \right)$$

In the limit $T_{DR}=0$, i.e. the time between the donor and the recipient’s measurement is small, $\boldsymbol{\delta}_{DR}$ is independent of$\boldsymbol{g}_{D}$ and heritability as estimated by parent offspring regression will be identical to heritability as estimated by fraction of phenotypic variance explained by genetic variance.

In the general case $T_{DR}>0$, however, $\boldsymbol{g}_{D}$ and $\boldsymbol{\delta}_{DR}$ will be correlated, so the covariance $\mathrm{cov}\left[ \boldsymbol{g}_{D},\boldsymbol{\delta}_{DR} \right]$ cannot be neglected. Specifically, we show below that this covariance is expected to be negative, such that under the OU model the donor-recipient regression coefficient will tend to be smaller than heritability as estimated by the fraction of phenotypic variance explained by genetic variance.

Let us compute further the covariance:

$$\mathrm{cov}\left[ \boldsymbol{g}_{D},\boldsymbol{\delta}_{DR} \right]=\bar{\boldsymbol{g}_{D}\boldsymbol{\delta}_{DR}}-\bar{\boldsymbol{g}_{D}} \bar{\boldsymbol{\delta}_{DR}}$$

Where the bar denotes average over the population. The covariance is a random variable. The two stochastic variables $\boldsymbol{g}_{D}$ and $\boldsymbol{\delta}_{DR}$ follow normal distributions:

$$g_{D}^{i}\mathcal{\sim N}\left( \left( 1-e^{-\alpha t_{i}} \right)\theta+e^{-\alpha t_{i}}g_{a}, \frac{\sigma^{2}}{2\alpha}\left( 1-e^{-2\alpha t_{i}} \right)+\epsilon^{2} \right)$$

$$\delta_{DR}^{i}\mathcal{\sim N}\left( \left( 1-e^{-\alpha T_{DR}} \right)\theta+e^{-\alpha T_{DR}}g_{D}^{i}, \frac{\sigma^{2}}{2\alpha}\left( 1-e^{-2\alpha T_{DR}} \right) \right)$$

The expectation of the covariance over many realizations of the stochastic evolutionary process (denoted by $E\left[ . \right]$) can be decomposed into:

$$E\left[ \bar{\boldsymbol{g}_{D}} \right]=\frac{1}{N}\sum_{i=1}^{N} (\left( 1-e^{-\alpha t_{i}} \right)\theta+e^{-\alpha t_{i}}g_{a})$$

$$E\left[ \bar{\boldsymbol{\delta}_{DR}} \right]=\left( 1-e^{-\alpha T_{DR}} \right)\theta+\left( e^{-\alpha T_{DR}}-1 \right)E\left[ \bar{\boldsymbol{g}_{D}} \right]$$

$$E\left[ \bar{\boldsymbol{g}_{D}\boldsymbol{\delta}_{DR}} \right]=\frac{1}{N}\sum_{i=1}^{N} E\left[ g_{D}^{i}\delta_{DR}^{i} \right]$$

$E\left[ g_{D}^{i}\delta_{DR}^{i} \right]$ is the expectation of the product $g_{D}^{i}\delta_{DR}^{i}$. Assuming the tree is ultrametric, we have $t_{i}=t$ for all $t$, and consequently $E\left[ \bar{\boldsymbol{g}_{D}} \right]=\left( 1-e^{-\alpha t} \right)\theta+e^{-\alpha t}g_{a}$, and

$$E\left[ \bar{\boldsymbol{\delta}_{DR}} \right]=e^{-\alpha\left( t+T_{DR} \right)}\left( e^{\alpha T_{DR}}-1 \right)(g_{a}-\theta)$$

In that case, the expectation of the covariance is:

$$E\left[ \mathrm{cov}\left[ \boldsymbol{g}_{D},\boldsymbol{\delta}_{DR} \right] \right]=-\frac{\sigma^{2}}{2\alpha} (1-e^{-\alpha T_{DR}}) \left( \frac{2\alpha\epsilon^{2}}{\sigma^{2}}+1-e^{-2\alpha t} \right)$$

which is 0 when $T_{DR}=0$ and negative when $T_{DR}>0$. Thus, on average, heritability as estimated by donor-recipient regression will be smaller than heritability as estimated by fraction of phenotypic variance explained by genetic variance (result iii).

References

1. Lynch M, Walsh B. Genetics and analysis of quantitative traits. Genetics and analysis of quantitative traits. 1998. 1-980 p.

2. Fraser C, Lythgoe K, Leventhal GE, Shirreff G, Hollingsworth TD, Alizon S, et al. Virulence and pathogenesis of HIV-1 infection: an evolutionary perspective. Science. 2014;343(6177):1243727.

3. Shirreff G, Alizon S, Cori A, Günthard HF, Laeyendecker O, van Sighem A, et al. How effectively can HIV phylogenies be used to measure heritability? Evol Med public Heal. 2013;2013(1):209–24.

4. Mitov V, Stadler T. The heritability of pathogen traits-definitions and estimators. bioRxiv. 2016;
