# Supplementary material for: Viral genetic variation accounts for a third of variability in HIV-1 set-point viral load in Europe
Source: PLoS Biol. 2017 Jun 12;15(6):e2001855. doi: 10.1371/journal.pbio.2001855 (PMC5467800; doi:10.1371/journal.pbio.2001855)
Supplement: S3 Text — (DOCX) [file pbio.2001855.s013.docx]

# Members of the cohorts:

## Swiss HIV cohort:

The member of the Swiss HIV Cohort are: Aubert V, Battegay M, Bernasconi E, Böni J, Braun DL, Bucher HC, Burton-Jeangros C, Calmy A, Cavassini M, Dollenmaier G, Egger M, Elzi L, Fehr J, Fellay J, Furrer H (Chairman of the Clinical and Laboratory Committee), Fux CA, Gorgievski M, Günthard H (President of the SHCS), Haerry D (deputy of “Positive Council”), Hasse B, Hirsch HH, Hoffmann M, Hösli I, Kahlert C, Kaiser L, Keiser O, Klimkait T, Kouyos R, Kovari H, Ledergerber B, Martinetti G, Martinez de Tejada B, Marzolini C, Metzner K, Müller N, Nadal D, Nicca D, Pantaleo G, Rauch A (Chairman of the Scientific Board), Regenass S, Rudin C (Chairman of the Mother & Child Substudy), Schöni-Affolter F (Head of Data Centre), Schmid P, Speck R, Stöckle M, Tarr P, Trkola A, Vernazza P, Weber R, Yerly S.

## ATHENA cohort (The Netherlands):

CLINICAL CENTRES:

* denotes site coordinating physician

**Academic Medical Centre of the University of Amsterdam:** *HIV treating physicians:* J.M. Prins*, T.W. Kuijpers, H.J. Scherpbier, J.T.M. van der Meer, F.W.M.N. Wit, M.H. Godfried, P. Reiss, T. van der Poll, F.J.B. Nellen, S.E. Geerlings, M. van Vugt, D. Pajkrt, W.J. Wiersinga, M. van der Valk, A. Goorhuis, J.W. Hovius. *HIV nurse consultants:* M.A.H. Bijsterveld, J. van Eden, A.M.H. van Hes, M. Mutschelknauss, H.E. Nobel, F.J.J. Pijnappel, A.M. Weijsenfeld. *HIV clinical virologists/chemists:* S. Jurriaans, N.K.T. Back, H.L. Zaaijer, B. Berkhout, M.T.E. Cornelissen, C.J. Schinkel, X.V. Thomas. **Admiraal De Ruyter Ziekenhuis, Goes:** *HIV treating physicians:* M. van den Berge, A. Stegeman. *HIV nurse consultants:* S. Baas, L. Hage de Looff. *HIV clinical virologists/chemists:* B Wintermans, J Veenemans. **Catharina Ziekenhuis, Eindhoven:** *HIV treating physicians:* M.J.H. Pronk*, H.S.M. Ammerlaan. *HIV nurse consultants:* E.S. de Munnik, E. van Beek. *HIV clinical virologists/chemists:* A.R. Jansz, J. Tjhie, M.C.A. Wegdam, B. Deiman, V. Scharnhorst. **Elisabeth-TweeSteden Ziekenhuis, Tilburg:** *HIV treating physicians:* M.E.E. van Kasteren*, A.E. Brouwer. *HIV nurse consultants:* R. van Erve, B.A.F.M. de Kruijf-van de Wiel, S.Keelan-Pfaf, B. van der Ven. *Data collection:* B.A.F.M. de Kruijf-van de Wiel, B. van der Ven. *HIV clinical virologists/chemists:* A.G.M. Buiting, P.J. Kabel, D.Versteeg. **Emma Kinderziekenhuis:** *HIV nurse consultants:* A. van der Plas, A.M. Weijsenfeld. **Erasmus MC, Rotterdam:** *HIV treating physicians:* M.E. van der Ende*, H.I. Bax, E.C.M. van Gorp, J.L. Nouwen, B.J.A. Rijnders, C.A.M. Schurink, A. Verbon, T.E.M.S. de Vries-Sluijs. *HIV nurse consultants:* N. Bassant, J.E.A. van Beek, M. Vriesde, L.M. van Zonneveld. *Data collection:* H.J. van den Berg-Cameron, F.B. Bruinsma-Broekman, J. de Groot, M. de Zeeuw-de Man. *HIV clinical virologists/chemists:* C.A.B. Boucher, M.P.G Koopmans, J.J.A van Kampen, S.D. Pas. **Erasmus MC–Sophia, Rotterdam:** *HIV treating physicians:* G.J.A. Driessen, A.M.C. van Rossum. *HIV nurse consultants:* L.C. van der Knaap, E. Visser. **Flevoziekenhuis, Almere:** *HIV treating physicians:* J. Branger*, A. Rijkeboer-Mes. *HIV nurse consultant and data collection:* C.J.H.M. Duijf-van de Ven. **HagaZiekenhuis, Den Haag:** *HIV treating physicians:* E.F. Schippers*, C. van Nieuwkoop. *HIV nurse consultants:* J.M. van IJperen, J. Geilings. *Data collection:* G. van der Hut. *HIV clinical virologist/chemist:* P.F.H. Franck. **HIV Focus Centrum (DC Klinieken):** *HIV treating physicians:* A. van Eeden*. *HIV nurse consultants:* W. Brokking, M. Groot, L.J.M. Elsenburg. *HIV clinical virologists/chemists:* M. Damen, I.S. Kwa. **Isala, Zwolle:** *HIV treating physicians:* P.H.P. Groeneveld*, J.W. Bouwhuis. *HIV nurse consultants:* J.F. van den Berg, A.G.W. van Hulzen. *Data collection:* G.L. van der Bliek, P.C.J. Bor. *HIV clinical virologists/chemists:* P. Bloembergen, M.J.H.M. Wolfhagen, G.J.H.M. Ruijs. **Leids Universitair Medisch Centrum, Leiden:** *HIV treating physicians:* F.P. Kroon*, M.G.J. de Boer, H. Jolink, A.M. Vollaard. *HIV nurse consultants:* W. Dorama, N. van Holten. *HIV clinical virologists/chemists:* E.C.J. Claas, E. Wessels. **Maasstad Ziekenhuis, Rotterdam:** *HIV treating physicians:* J.G. den Hollander*, K. Pogany, A. Roukens. *HIV nurse consultants:* M. Kastelijns, J.V. Smit, E. Smit, D. Struik-Kalkman, C. Tearno. *Data collection:* M. Bezemer, T. van Niekerk. *HIV clinical virologists/chemists:* O. Pontesilli. **Maastricht UMC+, Maastricht:** *HIV treating physicians:* S.H. Lowe*, A.M.L. Oude Lashof, D. Posthouwer. *HIV nurse consultants:* R.P. Ackens, J. Schippers, R. Vergoossen. *Data collection:* B. Weijenberg-Maes. *HIV clinical virologists/chemists:* I.H.M. van Loo, T.R.A. Havenith. **MCH-Bronovo, Den Haag:** *HIV treating physicians:* E.M.S. Leyten*, L.B.S. Gelinck. *HIV nurse consultants:* A.Y. van Hartingsveld, C. Meerkerk, G.S. Wildenbeest. *HIV clinical virologists/chemists:* J.A.E.M. Mutsaers, S.Q. van Veen. **MC Slotervaart, Amsterdam:** *HIV treating physicians:* J.W. Mulder*, S.M.E. Vrouenraets, F.N. Lauw. *HIV nurse consultants:* M.C. van Broekhuizen, H. Paap, D.J. Vlasblom. *HIV clinical virologists/chemists:* P.H.M. Smits. **MC Zuiderzee, Lelystad:** *HIV treating physicians:* S. Weijer*, R. El Moussaoui. *HIV nurse consultant:* A.S. Bosma. **Medisch Centrum Leeuwarden, Leeuwarden:** *HIV treating physicians:* M.G.A.van Vonderen*, D.P.F. van Houte, L.M. Kampschreur. *HIV nurse consultants:* K. Dijkstra, S. Faber. *HIV clinical virologists/chemists:* J Weel. **Medisch Spectrum Twente, Enschede:** *HIV treating physicians:* G.J. Kootstra*, C.E. Delsing. *HIV nurse consultants:* M. van der Burg-van de Plas, H. Heins. *Data collection:* E. Lucas. **Noordwest Ziekenhuisgroep, Alkmaar:** *HIV treating physicians:* W. Kortmann*, G. van Twillert*, J.W.T. Cohen Stuart, B.M.W. Diederen, R. Renckens. *HIV nurse consultant and data collection:* D. Ruiter-Pronk, F.A. van Truijen-Oud. *HIV clinical virologists/chemists:* W. A. van der Reijden, R. Jansen. **OLVG, Amsterdam:** *HIV treating physicians:* K. Brinkman*, G.E.L. van den Berk, W.L. Blok, P.H.J. Frissen, K.D. Lettinga W.E.M. Schouten, J. Veenstra. *HIV nurse consultants:* C.J. Brouwer, G.F. Geerders, K. Hoeksema, M.J. Kleene, I.B. van der Meché, M. Spelbrink, H. Sulman, A.J.M. Toonen, S. Wijnands. *HIV clinical virologists:* M. Damen, D. Kwa. *Data collection:* E. Witte. **Radboudumc, Nijmegen:** *HIV treating physicians:* R. van Crevel*, M. Keuter, A.J.A.M. van der Ven, H.J.M. ter Hofstede, A.S.M. Dofferhoff. *HIV nurse consultants:*M. Albers, K.J.T. Grintjes-Huisman, B.J. Zomer, A. Hairwassers. *HIV clinical virologists/chemists:*J. Rahamat-Langendoen. *HIV clinical pharmacology consultant:*D. Burger. **Rijnstate, Arnhem:** *HIV treating physicians:* E.H. Gisolf*, R.J. Hassing, M. Claassen. *HIV nurse consultants:* G. ter Beest, P.H.M. van Bentum, N. Langebeek. *HIV clinical virologists/chemists:* R. Tiemessen, C.M.A. Swanink. **Spaarne Gasthuis, Haarlem:** *HIV treating physicians:* S.F.L. van Lelyveld*, R. Soetekouw. *HIV nurse consultants:* L.M.M. van der Prijt, J. van der Swaluw. *Data collection:* N. Bermon. *HIV clinical virologists/chemists:* W.A. van der Reijden, R. Jansen, B.L. Herpers, D.Veenendaal. **Medisch Centrum Jan van Goyen, Amsterdam:** *HIV treating physicians:* D.W.M. Verhagen. *HIV nurse consultants:* M. van Wijk. **Universitair Medisch Centrum Groningen, Groningen:** *HIV treating physicians:* W.F.W. Bierman*, M. Bakker, J. Kleinnijenhuis, E. Kloeze, H. Scholvinck, Y. Stienstra, C.L. Vermont, K.R. Wilting. *HIV nurse consultants:* A. Boonstra, H. de Groot-de Jonge, P.A. van der Meulen, D.A. de Weerd. *HIV clinical virologists/chemists:* H.G.M. Niesters, C.C. van Leer-Buter, M. Knoester. **Universitair Medisch Centrum Utrecht, Utrecht:** *HIV treating physicians:* A.I.M. Hoepelman*, J.E. Arends, R.E. Barth, A.H.W. Bruns, P.M. Ellerbroek, T. Mudrikova, J.J. Oosterheert, E.M. Schadd, M.W.M. Wassenberg, M.A.D. van Zoelen. *HIV nurse consultants:* K. Aarsman, D.H.M. van Elst-Laurijssen, E.E.B. van Oers-Hazelzet. *Data collection:* M. van Berkel. *HIV clinical virologists/chemists:* R. Schuurman, F. Verduyn-Lunel, A.M.J. Wensing. **VUmc, Amsterdam:** *HIV treating physicians:* E.J.G. Peters*, M.A. van Agtmael, M. Bomers, J. de Vocht. *HIV nurse consultants:* M. Heitmuller, L.M. Laan. *HIV clinical virologists/chemists:* C.W. Ang, R. van Houdt, A.M. Pettersson, C.M.J.E. Vandenbroucke-Grauls. **Wilhelmina Kinderziekenhuis, UMCU, Utrecht:** *HIV treating physicians:* S.P.M. Geelen, T.F.W. Wolfs, L.J. Bont. *HIV nurse consultants:* N. Nauta.

COORDINATING CENTRE:

*Director:* P. Reiss. *Data analysis:* D.O. Bezemer, A.I. van Sighem, C. Smit, F.W.M.N. Wit, T.S. Boender. *Data management and quality control:* S. Zaheri, M. Hillebregt, A. de Jong. *Data monitoring:* D. Bergsma, A. de Lang, S. Grivell, A. Jansen, M.J. Rademaker, M. Raethke, R. Meijering, S. Schnörr. *Data collection:* L. de Groot, M. van den Akker, Y. Bakker, E. Claessen, A. El Berkaoui, J. Koops, E. Kruijne, C. Lodewijk, L. Munjishvili, B. Peeck, C. Ree, R. Regtop, Y. Ruijs, T. Rutkens, L. van de Sande, M. Schoorl, A. Timmerman, E. Tuijn, L. Veenenberg, S. van der Vliet, A. Wisse, T. Woudstra. *Patient registration:* B. Tuk.

## Antwerp cohort (Belgium)

Data extraction for the Antwerp Cohort is done by Maartje Van Frankenhuijsen, MD.

## PRIMO cohort (France):

**Région Sud–Est:**

- Thierry ALLEGRE, Centre hospitalier général d’Aix en Provence, Service d’Hématologie
- Djamila MAKHLOUFI, Jean-Michel LIVROZET, Pierre CHIARELLO, Mayhieu GODINOT, Florence BRUNEL-DALMAS, Sylvie GIBERT, Hôpital Edouard Herriot de Lyon, Immunologie Clinique
- Christian TREPO, Dominique PEYRAMOND, Patrick MIAILHES, Joseph KOFFI, Valérie THOIRAIN, Corinne BROCHIER, Thomas BAUDRY, Sylvie PAILHES, Lyon La Croix Rousse, Services d’Hépato-Gastroentérologie et des Maladies Infectieuses
- Alain LAFEUILLADE, Gisèle PHILIP, Gilles HITTINGER, Assi ASSI, Véronique LAMBRY, Hôpital Font-Pré de Toulon, Médecine Interne, Hémato-Infectiologie
- Eric ROSENTHAL, Alissa NAQVI, Brigitte DUNAIS, Eric CUA, Christian PRADIER, Jacques DURANT, Aline JOULIE, Hôpital L’Archet, Nice, Service de Médecine Interne, Maladies Infectieuses et Tropicales
- Denis QUINSAT, Serge TEMPESTA, Centre Hospitalier d’Antibes, Service de Médecine Interne
- Isabelle RAVAUX, Hôpital de la Conception de Marseille, Service des Maladies Infectieuses
- Isabelle POIZOT MARTIN, Olivia FAUCHER, Nicolas CLOAREC, Hôpital Sainte Marguerite de Marseille, Unité d'Hématologie
- Hélène CHAMPAGNE, Centre Hospitalier de Valence, Maladies Infectieuses et Tropicales
- Gilles PICHANCOURT, Centre Hospitalier Henri Duffaut d’Avignon, Service Hématologie Maladies Infectieuses

**Région Sud-Ouest:**

- Philippe MORLAT, Thierry PISTONE, Fabrice BONNET, Patrick MERCIE, Isabelle FAURE, Mojgan HESSAMFAR, Denis MALVY, Denis LACOSTE, Marie-Carmen PERTUSA, Marie-Anne VANDENHENDE, Noëlle  BERNARD, François PACCALIN, Cédric MARTELL, Julien ROGER-SCHMELZ, Marie-Catherine RECEVEUR, Pierre DUFFAU, Denis DONDIA, Emmanuel RIBEIRO, Sabrina CALTADO, Hôpital Saint André de Bordeaux, Médecine Interne
- Didier NEAU, Michel DUPONT; Hervé DUTRONC, Frédéric DAUCHY, Charles CAZANAVE, Thierry PISTONE, Marc-Olivier VAREIL, Thierry PISTONE, Gaétane WIRTH, Séverine LE PUIL, Hôpital Pellegrin de Bordeaux, Maladies Infectieuses.
- Jean-Luc PELLEGRIN, Isabelle RAYMOND, Jean-François VIALLARD, Severin CHAIGNE DE LALANDE, Hôpital Haut Lévèque de Bordeaux, Médecine Interne et Maladies Infectieuses
- Daniel GARIPUY, Hôpital Joseph Ducuing de Toulouse, Médecine Interne
- Pierre DELOBEL, Martine OBADIA, Lise CUZIN, Muriel ALVAREZ, Noemie BIEZUNSKI, Lydie PORTE, Patrice MASSIP, Alexa DEBARD, Florence BALSARIN, Myriam LAGARRIGUE, Hôpital Purpan de Toulouse, SMIT-CISIH
- François PREVOTEAU DU CLARY, Christian AQUILINA, Cité de la santé Toulouse
- Jacques REYNES, Vincent BAILLAT, Corinne MERLE, Vincent LEMOING, Nadine ATOUI, Alain MAKINSON, Jean Marc JACQUET, Christina PSOMAS, Christine TRAMONI, Hôpital Gui de Chauliac de Montpellier, Service des Maladies Infectieuses et Tropicales
- Hugues AUMAITRE, Mathieu SAADA, Marie MEDUS, Martine MALET, Aurélia EDEN, Ségolène NEUVILLE, Milagros FERREYRA, Martine MALET, Hôpital Saint Jean de Perpignan, Service des Maladies Infectieuses
- Albert SOTTO, Claudine BARBUAT, Isabelle ROUANET, Didier LEUREILLARD, Jean-Marc MAUBOUSSIN, Catherine LECHICHE, Régine DONSESCO, CHU de Nîmes-Caremeau, Service des Maladies Infectieuses et Tropicales.

**Antilles:**

- André CABIE, Sylvie ABEL, Sandrine PIERRE-FRANCOIS, Anne-Sophie BATALA, Christophe CERLAND, Camille RANGOM, Nadine THERESINE, CHU Fort de France, Hôpital de Jour
- Bruno HOEN, Isabelle LAMAURY, Isabelle FABRE, Kinda SCHEPERS, Elodie CURLIER, Rachida OUISSA, CHU de Pointe à Pitre/ABYMES, Service de Dermatologie / Maladies Infectieuses

**Région Est:**

- Geneviève BECK-WIRTH, Catherine MICHEL, Charles BECK, Jean-Michel HALNA, Jakub KOWALCZYK Meryem BENOMAR, Hôpital Emile Muller de Mulhouse, Hématologie Clinique
- Christine DROBACHEFF-THIEBAUT, Catherine CHIROUZE, Jean-François FAUCHER, François PARCELIER, Adeline FOLTZER, Cécile HAFFNER-MAUVAIS, Mathieu HUSTACHE MATHIEU, Aurélie PROUST - Hôpital St Jacques de Besançon, Service des Maladies Infectieuses et de Dermatologie
- Lionel PIROTH, Pascal CHAVANET, Michel DUONG, Marielle BUISSON, Anne WALDNER, Sophie MAHY, Sandrine GOHIER, Delphine CROISIER, Hôpital du Bocage de Dijon, Service des Maladies Infectieuses
- Thierry MAY, Mikael DELESTAN, Marie ANDRE, CHU de Vandoeuvre-lès-Nancy, Hôpital de Brabois, Service des Maladies Infectieuses et Tropicales
- Mahsa MOHSENI ZADEH, Martin MARTINOT, Béatrice ROSOLEN, Anne PACHART, Hôpital Louis PASTEUR de Colmar, Service d’Immunologie Clinique
- Benoît MARTHA, Noëlle JEUNET, Centre Hospitalier William Morey de Chalon Sur Saône, Service de Médecine Interne
- David Rey, Christine CHENEAU, Maria PARTISANI, Michèle PRIESTER, Claudine BERNARD-HENRY, Maria PARTISANI, Marie-Laure BATARD, Patricia FISCHER, Service le Trait d’Union, Hôpitaux Universitaires de Strasbourg
- Jean-Luc BERGER, Isabelle KMIEC, Hôpital Robert Debré, Service des Maladies Infectieuses, Reims.

**Région Nord:**

- Olivier ROBINEAU, Thomas HULEUX, Faïza AJANA, Isabelle ALCARAZ, Christophe ALLIENNE, Véronique BACLET, Agnès MEYBECK, Michel VALETTE, Nathalie VIGET, Christophe ALLIENNE, Emmanuelle AISSI, Raphael BIEKRE, Pauline CORNAVIN, Centre Hospitalier DRON de Tourcoing, Service de Maladies Infectieuses
- Dominique MERRIEN, Jean-Christophe SEGHEZZI, Moise MACHADO, Centre Hospitalier de Compiègne, Service de Médecine Interne
- Georges DIAB, C H de la Haute Vallée de l’Oise de Noyon, Service de Médecine

**Région Ouest:**

- François RAFFI, Bénédicte BONNET, Clotilde ALLAVENA, Olivier GROSSI, Véronique RELIQUET, Eric BILLAUD, Cecile BRUNET, Sabelline BOUCHEZ, Pascale MORINEAU-LE HOUSSINE, Fabienne SAUSER, David BOUTOILLE, Michel BESNIER, Hervé HUE, Nolwenn Hall, Delphine BROSSEAU, Hôtel-Dieu de Nantes, CISIH Médecine Interne
- Faouzi SOUALA, Christian MICHELET, Pierre TATTEVIN, Cédric ARVIEUX, Matthieu REVEST, Helene LEROY, Jean-Marc CHAPPLAIN, Matthieu DUPONT, Fabien FILY, Solène PATRA-DELO, Céline LEFEUVRE, CHRU Pontchaillou de Rennes, Clinique des Maladies Infectieuses
- Louis BERNARD, Frédéric BASTIDES, Pascale NAU, Hôpital Bretonneau de Tours, Service des maladies Infectieuses
- Renaud VERDON, Arnaud DE LA BLANCHARDIERE, Anne MARTIN, Philippe FERET, CH régional Côte de Nacre de Caen, Service de Maladies Infectieuses
- Loïk GEFFRAY, Hôpital Robert Bisson de Lisieux, Service de Médecine Interne
- Corinne DANIEL, Jennifer ROHAN, Centre Hospitalier La Beauchée de Saint-Brieuc, Médecine Interne et Maladies Infectieuses
- Pascale FIALAIRE, Jean Marie CHENNEBAULT, Valérie Rabier, Pierre ABGUEGUEN, Sami REHAIEM, Centre Hospitalier Régional d’Angers, Service des Maladies Infectieuses
- Odile LUYCX, Mathilde NIAULT, Philippe MOREAU, Centre Hospitalier Bretagne Sud de Lorient, Service d’Hématologie
- Yves POINSIGNON, Marie GOUSSEF, Virginie MOUTON- RIOUX, Centre Hospitalier Bretagne Atlantique de Vannes, Service de Medecine Interne et Maladies Infectieuses
- Dominique HOULBERT, Sandrine ALVAREZ-HUVE, Frédérique BARBE, Sophie HARET, Centre Hospitalier d’Alençon, Médecine 2
- Philippe PERRE,Sophie LEANTEZ-NAINVILLE, Jean-Luc ESNAULT, Thomas GUIMARD, Isabelle SUAUD, Centre Hospitalier Départemental de La Roche sur Yon, Service de Médecine
- Jean-Jacques GIRARD, Véronique SIMONET, Hôpital de Lôches, Service de Médecine Interne
- Yasmine DEBAB, CHU Charles Nicolle de Rouen, Maladies Infectieuses et Tropicales
- Jean-Luc SCHMIT, CHU d’Amiens, Service des Maladies Infectieuses.

**Région Centre:**

- Christine JACOMET, Hôpital Gabriel-Montpied de Clermont Ferrand, Service des Maladies Infectieuses et Tropicales
- Pierre WEINBERCK, Claire GENET, Pauline PINET, Sophie DUCROIX, Hélène DUROX, Éric DENES, Hôpital DUPUYTREN de Limoges, Maladies Infectieuses et Tropicales
- Bruno ABRAHAM, Centre Hospitalier de Brive, Departement de maladies Infectieuses
- Florence GOURDON, Centre Hospitalier de Vichy, Service de Médecine Interne
- Odile ANTONIOTTI, Centre Hospitalier de Montluçon, Dermatologie

**Paris:**

- Jean-Michel MOLINA, Samuel FERRET, Caroline LASCOUX-COMBE, Matthieu LAFAURIE, Nathalie COLIN DE VERDIERE, Diane PONSCARME, Nathalie DE CASTRO, Alexandre ASLAN, Willy ROZENBAUM, Claire PINTADO, François CLAVEL, Olivier TAULERA, Caroline GATEY, Anne-Lise MUNIER, Sandrine GAZAIGNE, Pauline PENOT, Guillaume CONORT, Nathalie LEROLLE, Anne LEPLATOIS, Stéphanie BALAUSINE, Jeannine DELGADO, Hôpital Saint Louis de Paris, Service des Maladies Infectieuses et Tropicales
- Julie TIMSIT, Magda TABET, Hôpital Saint Louis de Paris, Clinique MST
- Laurence GERARD, Hôpital Saint Louis de Paris, Service d'Immunologie Clinique
- Pierre-Marie GIRARD, Odile PICARD, Jürgen TREDUP, Diane BOLLENS, Nadia VALIN, Pauline CAMPA, Julie BOTTERO, Benedicte LEFEBVRE, Muriel TOURNEUR, Laurent FONQUERNIE, Charlotte WEMMERT, Jean-Luc LAGNEAU Hôpital Saint Antoine de Paris , Service des Maladies Infectieuses et Tropicales
- Yazdan YAZDANPANAH, Bao PHUNG, Adriana PINTO, Dorothée VALLOIS, Ornella CABRAS, Françoise LOUNI, G. Hospitalier Bichat-Claude Bernard de Paris, Service de Maladies Infectieuses et Tropicales
- Gilles PIALOUX, Thomas LYAVANC, Laurence SLAMA, Valérie BERREBI, Julie CHAS, Thomas L’YAVANAC, Sophie LENAGAT, Hopital Tenon de Paris, Service des Maladies Infectieuses
- Agathe RAMI, Myriam DIEMER, Maguy PARRINELLO, Audrey DEPOND, Hôpital Lariboisière de Paris, Service de Médecine InterneA
- Dominique SALMON, Loïc GUILLEVIN, Tassadit TAHI, Linda BELARBI, Pierre LOULERGUE, Olivier ZAK DIT ZBAR, Odile LAUNAY, Benjamin SILBERMANN, Catherine LEPORT, Laura ALAGNA, Marie-Pierre PIETRI, G. H. Cochin de Paris, Département de Médecine Interne
- Anne SIMON, Manuela BONMARCHAND, Naouel AMIRAT, François PICHON, Myriam KIRSTETTER, G. H. Pitié-Salpétrière de Paris, Service de Médecine Interne
- Christine KATLAMA, Marc Antoine VALANTIN, Roland TUBIANA, Fabienne CABY, Luminita SCHNEIDER, Nadine KTORZA, Ruxandra CALIN, Audrey MERLET, Saadia BEN ABDALLAH, G. H. Pitié-Salpétrière de Paris, Service des Maladies Infectieuses
- Laurence WEISS, Martin BUISSON, Dominique BATISSE, Marina KARMOCHINE, Juliette PAVIE, Catherine MINOZZI, Didier JAYLE, Philippe CASTEL, Jean DEROUINEAU, Pascale KOUSIGNAN, Murielle ELIAZEVITCH, Isabelle PIERRE, Lio COLLIAS, Hôpital Européen Georges Pompidou de Paris, Service d'Immunologie Clinique
- Jean-Paul VIARD, Jacques GILQUIN, Alain SOBEL, Jade GHOSN, Blanka HADACEK, Nugyen THU-HUYN, Audrey MERLET, Lella NAIT-IGHIL, Agnes CROS, Aline MAIGNAN, Hôtel Dieu de Paris, Centre de Diagnostic et Thérapeutique
- Claudine DUVIVIER, Paul Henri CONSIGNY, Fanny LANTERNIER, Michka SHOAI-TEHRANI, Fatima TOUAM, Saadia JERBI, Centre Médical de l’Institut Pasteur de Paris, Service des Maladies Infectieuses
- Loïc BODARD, Corinne JUNG, Institut Mutualiste Montsouris de Paris, Département de Médecine Interne

**Région Parisienne:**

- Cécile GOUJARD, Yann QUERTAINMONT, Martin DURACINSKY, Olivier SEGERAL, Arnaud BLANC, Delphine PERETTI, Antoine CHERET, Christelle CHANTALAT, Marie Josée DULUCQ, Hôpital de Bicêtre, Médecine Interne
- Yves LEVY, Jean Daniel LELIEVRE, Anne Sophie LASCAUX, Cécile DUMONT, Hôpital Henri Mondor de Créteil, Immunologie Clinique
- François BOUE, Véronique CHAMBRIN, Imad KANSAU, Mariem RAHO-MOUSSA, Hôpital Antoine Béclère de Clamart, Médecine Interne et Immunologie Clinique
- Pierre DE TRUCHIS, Aurélien DINH, Benjamin DAVIDO, Dhiba MARIGOT, Huguette BERTHE, Hôpital Raymond Poincaré de Garches, Service des Maladies Infectieuses et Tropicales
- Alain DEVIDAS, Pierre CHEVOJON, Amélie CHABROL, Nouara AGHER, Hôpital de Corbeil-Essonnes, Service Hématologie
- Yvon LEMERCIER, Fabrice CHAIX, Isabelle TURPAULT, Centre Hospitalier Général de Longjumeau, Service de Médecine Interne
- Olivier BOUCHAUD, Patricia HONORE, Hôpital Avicenne de Bobigny, Maladies Infectieuses et Tropicales
- Elisabeth ROUVEIX, Evelyne REIMANN, Hôpital Ambroise Paré de Boulogne, Médecine Interne
- Alix GREDER BELAN, Claire GODIN COLLET, Safia SOUAK, Hôpital du Chesnay, CH Andre Mignot du Chesnay, Maladies Infectieuses et Tropicales
- Emmanuel MORTIER, Martine BLOCH, Anne-Marie SIMONPOLI, Véronique MANCERON, Isabelle CAHITTE, Emmanuel HIRAUX, Erik LAFON, François CORDONNIER ? Ai-feng ZENG, Hôpital Louis Mourier de Colombes, Médecine Interne
- David ZUCMAN, Catherine MAJERHOLC, Dominique BORNAREL, Hôpital Foch de Suresnes , Médecine Interne
- Agnès ULUDAG, Justine GELLEN-DAUTREMER, Agnès LEFORT, Christine BAZIN, Hôpital Beaujon de Clichy, Médecine Interne
- Vincent DANELUZZI, Juliette GERBE, Centre Hospitalier de Nanterre, Service de Médecine Interne
- Gérôme STIRNMAN, Mélissa COUPARD, Hôpital Jean Verdier de Bondy, Service de Médecine Interne, Unité de Maladies Infectieuses
- Olivier PATEY, Jonas BANTSIMBA, Sophie DELLLION, Pauline CARAUX PAZ, Benoit CAZENAVE, Laurent RICHIER, Centre Hospitalier Intercommunal de Villeneuve St Georges, Médecine Interne
- Valérie GARRAIT, Isabelle DELACROIX, Brigitte ELHARRAR, Laurent RICHIER, Centre Hospitalier Intercommunal de Créteil, Médecine Interne, Hépato-Gastroentérologie
- Daniel VITTECOQ, Claudine BOLLIOT, Hôpital de Bicêtre, Service de Maladies Infectieuses et Tropicales
- Annie LEPRETRE, Hôpital Simone Veil d’Eaubonne, Médecine 2, Consultation ESCALE
- Philippe GENET, Virginie MASSE, Juliette GERBE, Consultation d’Immuno/Hématologie d’Argenteuil
- Véronique PERRONE, Centre Hospitalier François Quesnay de Mantes La Jolie, Service des Maladies Infectieuses
- Jean-Luc BOUSSARD, Patricia CHARDON, Centre Hospitalier Marc Jacquet de Melun, Service de Médecine
- Eric FROGUEL, Phlippe SIMON, Sylvie TASSI, Hôpital de Lagny, Service de Médecine Interne

## **UK Register of HIV seroconverters:**

We would like to thank all the UK Register participants for allowing their routine clinical data to be included. We gratefully acknowledge the work of the members of the Steering Committee and colleagues at the clinical centres. Special thanks go to the following colleagues: Kristin Kuldanek, Scott Mullaney (St Mary’s Hospital, London), Carmel Young (Mortimer Market Centre, London), Antonella Zucchetti, Margaret-Ann Bevan (St Thomas’ Hospital, London), Sinead McKernan (Royal Victoria Hospital, Belfast), Emily Wandolo (King’s College Hospital, London), Celia Richardson, Elaney Youssef (Brighton and Sussex University Hospital), Pippa Green (Withington Hospital, Manchester), Sue Faulkner (Gloucester Royal Hospital), Rebecca Faville (Whittall Street Clinic, Birmingham), Sandra Herman, Christine Care (Royal Hallamshire Hospital, Sheffield), Helen Blackman (St Mary’s Hospital, Portsmouth), and Katharine Bellenger, Keith Fairbrother (Medical Research Council Clinical Trials Unit at UCL, London).

Members of the UK Register Steering Committee: Andrew Phillips (Chair), University College London (UCL), London; Abdel Babiker, UCL, London; Valerie Delpech, Public Health England, London; Sarah Fidler, St. Mary’s Hospital, London; Mindy Clarke, Brighton & Sussex University Hospitals NHS Trust, Brighton; Julie Fox, Guys and St Thomas’ NHS Trust/Kings College, London; Richard Gilson, West London Centre for Sexual Health, London; David Goldberg, Health Protection Scotland, Glasgow; David Hawkins, Chelsea & Westminster NHS Trust, London; Anne Johnson, UCL, London; Margaret Johnson, UCL and Royal Free NHS Trust, London; Ken McLean, West London Centre for Sexual Health, London; Eleni Nastouli, UCL, London; Frank Post, King’s College, London.

**The members of the UK Register of HIV seroconverters are:** N Kennedy, Monklands Hospital, Airdrie; J Pritchard, Ashford Hospital, Ashford; U Andrady, Ysbyty Gwynedd, Bangor; N Rajda, North Hampshire Hospital, Basingstoke; C Donnelly, S McKernan, Royal Victoria Hospital, Belfast; S Drake, G Gilleran, D White, Birmingham Heartlands Hospital, Birmingham; J Ross, J Harding, R Faville, Whittall Street Clinic, Birmingham; J Sweeney, P Flegg, S Toomer, Blackpool Victoria Hospital, Blackpool; H Wilding, R Woodward, Royal Bournemouth Hospital, Bournemouth; G Dean, C Richardson, N Perry, Royal Sussex County Hospital, Brighton; M Gompels, L Jennings, Southmead Hospital, Bristol; D Bansaal, Queen’s Hospital, Burton-Upon-Trent; M Browing, L Connolly, Cardiff Royal Infirmary, Cardiff; B Stanley, North Cumbria Acute Hospitals NHS Trust, Carlisle; S Estreich, A Magdy, St. Helier Hospital, Carshalton; CO’Mahony, Countess of Chester Hospital, Chester; P Fraser, Chesterfield & North Derbyshire Royal Hospital, Chesterfield; SPR Jebakumar, Essex County Hospital, Colchester; L David, Coventry & Warwickshire Hospital, Coventry; R Mette, Mayday University Hospital, Croydon; H Summerfield, Weymouth Community Hospital, Dorset; M Evans, Ninewells Hospital, Dundee; C White, University Hospital of North Durham, Durham; R Robertson, Muirhouse Medical Group, Edinburgh; C Lean, S Morris, Western General Hospital, Edinburgh; A Winter, Gartnavel General Hospital & Glasgow Royal Infirmary, Glasgow; S Faulkner, Gloucestershire Royal Hospital, Gloucester; B Goorney, Salford Hope Hospital, Greater Manchester; L Howard, Farnham Road Hospital, Guildford; I Fairley, C Stemp, Harrogate Hospital, Harrogate; L Short, Huddersfield Royal Infirmary, Huddersfield; M gomez, F young, St Mary’s Hospital Isle of Wight; M Roberts, S Green, Kidderminster General Hospital, Kidderminster; K Sivakumar, the Queen Elizabeth Hospital, King’s Lynn; J Minton, A Siminoni, Leeds General Infirmary, Leeds; J Calderwood, D Greenhough, J Minton, St. James’ Hospital, Leeds; C DeSouza, Lisa Muthern, C Orkin, Barts & the London NHS Trust, London; S Murphy, M Truvedi, Central Middlesex Hospital, London; K McLean, Charing Cross Hospital, London; D Hawkins, C Higgs, A Moyes, Chelsea & Westminster Hospital, London; S Antonucci, S McCormack, Dean Street Clinic, London; W Lynn, Ealing Hospital, London; M Bevan, J Fox, A Teague, Guy’s & St. Thomas NHS Trust, London; J Anderson, S Mguni, Homerton Hospital, London; F Post, L Campbell, E Wandolo King’s College Hospital, London; C Mazhude, H Russell, Lewisham University Hospital, London; R Gilson, G Carrick, C Young Mortimer Market Centre, London; J Ainsworth, A Waters, North Middlesex Hospital, London; P Byrne, M Johnson, Royal Free Hospital, London; London; S Fidler, K Kuldanek, S Mullaney, St. Mary’s Hospital, London; V Lawlor, R Melville, Whipps Cross Hospital, London; A Sukthankar, S Thorpe, Manchester Royal Infirmary, Manchester; C Murphy, E Wilkins, North Manchester General Hospital, Manchester; S Ahmad, P Green, Withington Hospital, Manchester; S Tayal, James Cook Hospital, Middlesbrough; E Ong, Newcastle General Hospital, Newcastle; J Meaden, Norfolk & Norwich University Hospital, Norwich; L Riddell, City Hospital, Nottingham; D Loay, K Peacock, George Eliot Hospital, Nuneaton; H Blackman, V Harindra, St. Mary’s Hospital, Portsmouth; AM Saeed, Royal Preston Hospital, Preston; S Allen, U Natarajan, East Surrey Hospital, Redhill; O Williams, Glan Clwyd District General, Rhyl; H Lacey, Baillie Street Health Centre, Rochdale; C Care, C Bowman, S Herman, Royal Hallamshire Hospital, Sheffield; SV Devendra, J Wither, Royal Shrewsbury Hospital, Shrewsbury; A Bridgwood, G Singh, North Staffordshire Hospital, Stoke-on-Trent; S Bushby, Sunderland Royal Hospital, Sunderland; D Kellock, S Young, King’s Mill Centre, Sutton-in-Ashfield; G Rooney, B Snart, the Great Western Hospital, Swindon; J Currie, M. Fitzgerald, Taunton & Somerset Hospital, Taunton; J Arumainayyagam, S Chandramani, Manor Hospital, Walsall; S Rajamanoharan, T Robinson, Watford General Hospital, Watford; M Roberts, Worcester Royal Infirmary, Worcester; O Williams, Maelor Hospital, Wrexham; B Taylor, Wycombe General Hospital, Wycombe; C Brewer, I Fairley, Monkgate Health Centre, York Hospital NHS Trust, York.
